# Liposomes and Other Nanocarriers for the Treatment of Acne Vulgaris: Improved Therapeutic Efficacy and Skin Tolerability

**DOI:** 10.3390/pharmaceutics16030309

**Published:** 2024-02-22

**Authors:** Nina Dragicevic, Howard I. Maibach

**Affiliations:** 1 Department of Pharmacy, Singidunum University, 11000 Belgrade, Serbia; 2 Dermatology Department, University of California, San Francisco, CA 94143-0989, USA; howard.maibach@ucsf.edu

**Keywords:** phospholipid-based vesicles, liposomes, invasomes, penetration-enhancer-containing vesicles (PEVs), ethosomes, acne vulgaris, adapalene, tretinoin, benzoyl peroxide

## Abstract

Acne vulgaris is a common dermatologic disorder that affects approximately 85% of teenagers, which significantly impacts the quality of life in adolescents. It is a chronic disease of the sebaceous follicles that is multifactorial in etiology. Topical treatment is the first choice for mild and moderate acne, while systemic therapy is reserved for severe and certain moderate cases. Topical treatments include retinoids (e.g., tretinoin and adapalene), antibiotics (e.g., clindamycine), and other agents (e.g., benzoyl peroxide and azelaic acid), often applied in combination. The mechanisms of action include antimicrobial, anti-inflammatory, and keratolytic activities, as well as sebum secretion reduction, and the normalization of follicular keratinization. However, these topical agents commonly induce side effects, such as dryness, burning, stinging, peeling, redness, erythema, and photosensitivity. Therefore, there is a need to reduce the side effects of anti-acne drugs, while maintaining or enhancing their therapeutic effectiveness. This article aims to comprehensively outline nanotechnology strategies, particularly the use of phospholipid-based nanocarriers like liposomes and related vesicles, to enhance therapeutic efficacy, skin tolerability, and patient compliance in the treatment of acne vulgaris. In addition, novel active ingredients encapsulated in vesicles beyond those recommended in official guidelines are discussed.

## 1. Introduction

**Figure 1:**
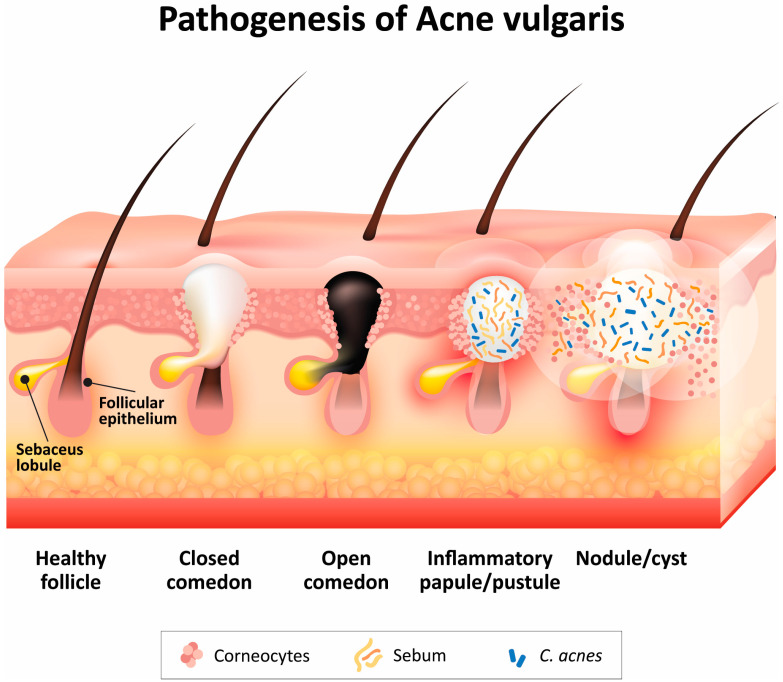
Schematic representation of pathogenesis of acne. Redrawn and modified figure from: https://www.freepik.com/free-photos-vectors/acne (accessed on 15 January 2024).

Acne vulgaris is the most common dermatologic disorder, which affects approximately 85% of teenagers and has a considerable impact on the quality of life of adolescents. It affects mostly the face, but upper chest, back, and shoulders may be also involved. Acne is a chronic disease of the sebaceous follicles that is multifactorial in etiology. It occurrs in pilosebaceous units consisting of the follicular canal with its rudimentary hair and a group of sebaceous glands that surround the opening of the follicle. Four main factors are involved in the development of acne: (1) abnormal follicular keratinocyte hyperproliferation, leading to the formation of a follicular plug, (2) increased sebum production within sebaceous follicles, (3) the proliferation of micro-organisms (e.g., *Cutibacterium acnes* (formerly *Proprionibacterium acnes*)) in the retained sebum, and (4) inflammation (Figure 1). In many patients, there is also a strong genetic element [1].

The symptoms of acne vulgaris are lesions, that include, in order of severity, comedones (open comedones—blackheads and closed comedones—whiteheads) as non-inflammatory lessions and papules, pustules, nodules, and cysts (Figure 1), all four of which are inflammatory lesions, which can develop into deep, purulent lesions in severe cases and lead to scarring [1].

There are many classifications of acne vulgaris. The United States Food and Drug Administratiom (US FDA) released a piece of guidance in May 2018 on treating acne vulgaris, providing recommendations based on clinical trial reviews. They proposed a global scale for acne severity assessment (Investigator Global Assessment (IGA)) with five severity grades (0–4) of lesions, where 0 indicates clear, 1 is almost clear, 2 is mild, 3 is moderate, and 4 indicates severe acne [2]. The updated Guideline of care for the management of acne vulgaris published by the American Academy of Dermatology (AAD) provides evidence-based recommendations to direct the clinical management of acne vulgaris in patients based on FDA-approved drugs. According to this guideline, among different available grading systems, the IGA indeed represents the most common used in the US, showing good agreement between the ratings of clinicians and patients [3]. The European guideline classifies acne into four categories: comedonal, papulopustular (mild–moderate and severe), and nodular (moderate and severe). Recommendations for treatment vary in strength based on severity, with alternatives for females [4]. The National Institute for Health and Care Excellence (NICE) guideline on acne vulgaris grades acne as “mild to moderate” or “moderate to severe” [5].

The topical route is preferred for the treatment of skin diseases like acne vulgaris as it delivers higher drug amounts directly to the infected area, reducing systemic effects. However, it may cause skin irritation. Oral antibiotics, like tetracyclines, are effective against inflammatory acne, but carry risks of severe adverse effects and antimicrobial resistance [6]. If treatment fails, the final drug is oral isotretinoin, which is highly effective in recalcitrant acne, but limited due to its moderate to serious side effects. Other treatments include chemical peels, laser therapy, and fat transplantation [7].

The route of drug administration depends on the severity of the disease. Topical treatment is the first choice in mild and moderate acne, whereas systemic therapy is used for severe and some moderate acne, acne that is resistant to topical treatment, and acne that covers large parts of the body surface. Topical therapy usually involves the use of retinoids, antibiotics, and combinations of topical anti-acne drugs, which usually irritate the skin. Systemic treatment includes oral antibiotics, oral retinoids (isotretinoin), and hormonal treatment [7].

The modes of action shown for active agents used in conventional acne therapy include antimicrobial activity (benzoyl peroxide and antibiotics), anti-inflammatory activity (retinoids), keratolytic activity (salicylic acid), the reduction of the secretion of sebum (retinoids), and the normalisation of follicular keratinisation (retinoids).

The aim of treating acne vulgaris is to inhibit the formation of new lesions, facilitate the healing of existing ones, and prevent permanent scarring, along with addressing potential negative impacts on psychosocial well-being [8].

The main aim of this article is to provide a comprehensive overview of nanotechnology strategies, i.e., the use of phospholipid-based vesicles (e.g., liposomes and others) for enhancing therapeutic efficacy and skin tolerability of drugs used in the treatment of acne vulgaris. Despite the existing reviews on liposomes and skin diseases, none comprehensively addresses the full spectrum of these phospholipid-based nanocarriers relevant to the topical treatment of acne. By consolidating the existing knowledge about liposomes, acne, as well as about different actives used for treating acne and identifying potential avenues for further exploration, we aim to facilitate advancements in the realm of topical acne treatment. In addition to the anti-acne agents recommended in the guidelines, new agents are also discussed in the review.

## 2. Limitations of Current Anti-Acne Formulations

Various medications and anti-acne formulations have been used to treat acne vulgaris. However, since the FDA approved tretinoin in 1971, retinoids alone or combined with other drugs have become the mainstay of acne treatment. Other drugs have also been used, but always in combination with retinoids. There are various formulations with retinoids on the market, which comprise gels, creams, foams, lotions, and solutions in a wide range of concentrations, from different manufacturers. Tretinoin has been used in gels (0.1%, 0.05%, 0.025%: Retin-A^®^, Atralin^®^), creams (0.1%, 0.05%, 0.02%, 0.025%: Retrieve^®^, Stevia-A^®^, Retin-A^®^, Renova^®^, Rejuva-A^®^), liquid formulations/solutions (0.05%: Retin-A^®^), lotions (0.05%: Altreno^®^), and microsphere gels (0.1%, 0.04%, 0.06%, 0.08%: Retin-A Micro^®^), etc. Adapalene is present on the market in the form of creams, gels, and lotions (0.1%: Differin^®^ gel, cream, and lotion, Epiduo^®^ gel—adapalene combined with BPO (0.1%/2.5% and 0.3%/2.5% (forte))). Tazarotene is a available in the form of topical gels (0.05% and 0.1%: Tazorac^®^, Zorac^®^), creams (0.05% and 0.1%: Tazorac^®^, Zorac^®^ and Avage^®^), and foams (0.1%: Fabior^®^). Isotretinoin is available on the market in the form of gels (0.05%: Isotrex^®^ gel, 0.05% isotretinoin, and 2% erythromycin: Isotrexin^®^ gel) [9]. The new retinoid trifarotene is present on the market in the form of a cream (0.005%: Aklief^®^) [10]. All these retinoids used in the topical treatment of acne have side effects i.e., skin irritation, including dryness, burning, stinging, peeling, redness, and erythema, as well as photosensitivity, etc. Despite the fact that adapalene (third-generation retinoid) shows superior skin tolerability over tretinoin (first generation) and tazarotene (third-generation), adapalene still shows skin irritation. Tazarotene was introduced to enhance therapeutic efficacy, and it succeded; however, it shows a greater level of burning, pruritus, and erythema than adapalene and tretinoin. The most recently approved retinoid trifarotene (fourth-generation) has been shown to be efficacious; however, it also failed to exclude local side effects, as skin irritation and pruritus have been reported [11]. Unfortunately, skin irritation, which is most prominent in the first weeks of treatment, is associated with poor tolerability and suboptimal adherence, which reduces the response to treatment.

Other drawbacks of retinoids include the instability of most retinoids at light and oxygen exposure, as well as their unfavorable physicochemical properties; i.e., they are lipophilic with poor water solubility, which makes their formulation into dosage forms difficult [9,11].

However, it is not only retinoids that lead to skin irritation and possess poor water solubility, but other anti-acne drugs, such as BPO (2.5–10%: lotions, foams, gels, shampoos), azelaic acid (10–20%: Skinoren^®^ cream, etc.), and salycilic acid also induce skin irritation, including redness, peeling, etc. [12], which should be minimized by new technologies. As to topical antibiotics, which, when used together with retinoids, represent a mainstay in acne treatment, when used for a longer time, they lead to the development of antibiotic-resistant strains of bacteria, making them less effective over time [13]. This represents a significant concern.

Therefore, it is necessary to apply strategies to improve the efficacy, safety, and tolerability of anti-acne drugs. New technologies are needed in order to reduce the undesired side effects of these drugs, such as skin irritation, thereby increasing patient compliance (and adherence) and the therapeutic effectiveness of anti-acne drugs. In addition, the solubility of poorly water-soluble drugs should be improved, for example, by the use of micronization, solid dispersions, cyclodextrins, and colloidal drug delivery systems (microemulsion, solid lipid nanoparticles, etc.) [14]. In addition, the photostability of anti-acne drugs should be enhanced, for example, by colloidal drug delivery systems [15].

Different colloidal delivery systems, i.e., nanocarriers frequently being used in dermal drug delivery [16], such as liposomes [17,18,19], niosomes [20], solid lipid nanoparticles, nanostructured lipid carriers [21], etc., could be used to address all the aforementioned drawbacks of anti-acne drugs. By encapsulating anti-acne drugs, these nanocarriers are able to minimize their side effects, and improve their tolerability and safety, as well as their therapeutic effectiveness. In addition, they enable their solubilization, which is needed during their formulation, increase their stability, and enable targeted delivery to the follicles and sebaceous glands, which has been documented in research studies [9,22,23], and will be discussed in the following sections.

## 3. Liposomes and Related Vesicles as Nanocarriers for Anti-Acne Drugs

**Figure 2:**
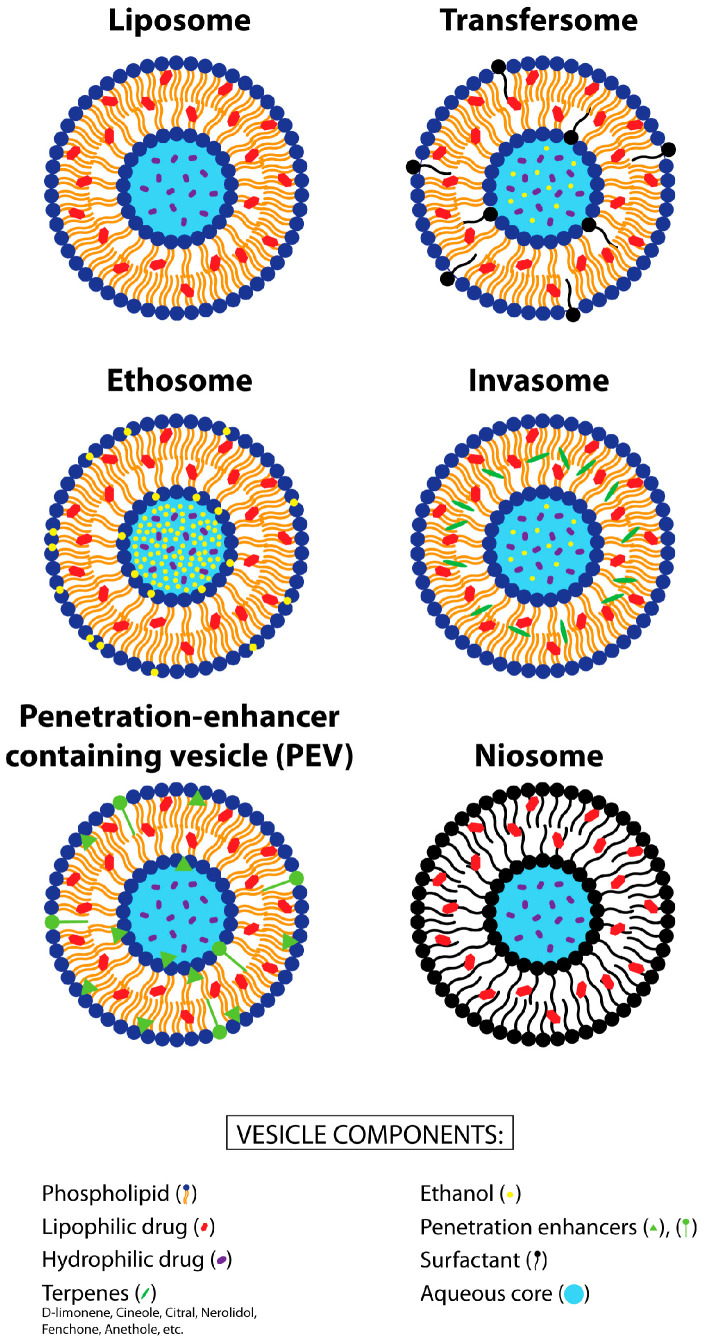
Schematic representation of different phospholipid-based vesicles and niosomes.

Liposomes have emerged as the pioneering nanocarriers for drug delivery across various administration routes, including skin penetration. Comprising different phospholipids, cholesterol, and an aqueous medium, liposomes are versatile colloidal particles. Commonly utilized natural phospholipids (PLs), such as phosphatidylcholine (PC), phosphatidylethanolamine (PE), phosphatidylserine (PS), phosphatidyl inositol (PI), and phosphatidyl glycerol (PG), are preferred for their cost-effectiveness and favorable toxicological profiles. In addition, phospholipids exhibit several advantageous characteristics, including biocompatibility, biodegradability, non-immunogenicity, and recognition as generally recognized as safe (GRAS) components by the FDA [15,24]. These attributes make phospholipid-based vesicles, i.e., liposomes, highly suitable as drug nanocarriers. Liposomes can encapsulate hydrophilic drugs within their aqueous compartments and incorporate lipophilic drugs within their lipid bilayers (Figure 2). Additionally, liposomes have the capacity to enhance the solubility of poorly water-soluble drugs, which is important since most drugs used for the treatment of acne are not hydrophilic, as well as to improve the chemical stability of drugs, particularly with regard to photostability [15,25].

Liposomes have found significant application in dermal and transdermal drug delivery, including their targeted delivery to skin appendages. In the context of dermal drug delivery, liposomes are employed to enhance drug penetration into the skin, ensuring localized therapeutic levels while minimizing percutaneous absorption [26,27,28]. Liposomes contribute to improved therapeutic efficacy [29,30] and the reduction of side effects [25,31] and serve as a local depot for the sustained release of dermally active components [32]. Conventional liposomes, composed of various combinations of PL, cholesterol, and water, represent the most extensively researched nanocarriers for dermal drug delivery.

In contrast, conventional liposomes are less effective for transdermal drug delivery, as they mainly remain confined to the stratum corneum (SC) and epidermis, and only smaller amounts penetrate into the dermis [32,33]. When a higher penetration-enhancing ability is necessary, regardless of whether dermal or transdermal drug delivery is the goal, then elastic/deformable liposomes have to be applied, as they are able to provide a higher drug penetration into or through the skin. Among different deformable vesicles, Transfersomes^®^ (Idea AG, München, Germany), ethosomes, invasomes, penetration-enhancer-containing vesicles (PEV), etc. [34,35,36,37,38,39,40,41], have been successfully employed. Transfersomes^®^ (a registered trademark of Idea AG, Germany) are the pioneering deformable vesicles, introduced by Cevc and colleagues [42]. These vesicles include PC along with edge activators like sodium cholate, polysorbate 80, or polysorbate 20, which induce deformability and enhance penetration [42]. Ethosomes are vesicles composed of phospholipids, ethanol, and water. Ethanol incorporation into lipid vesicles enhances membrane fluidity and elasticity, thereby improving percutaneous drug penetration [43]. Invasomes are vesicles that, in addition to PC and the aqueous phase, contain small amounts of ethanol and terpenes or terpene mixtures as penetration enhancers, which increase the vesicle bilayer fluidity and skin penetration of drugs [18]. Penetration-enhancer-containing vesicles (PEVs) consist of soybean PC and varying amounts of penetration enhancers such as 2-(2-ethoxyethoxy) ethanol (Transcutol^®^), capryl-caproyl macrogol 8-glyceride (Labrasol^®^), and others, which, together, contribute to their percutaneous penetration-enhancing ability [44,45]. Thus, all aforementioned elastic vesicles contain additives besides PL, i.e., edge activators, which impart elasticity to the vesicles’ bilayers to facilitate their penetration into/through the skin, thereby leading to the increased penetration of drugs into/through the skin.

Besides phospholipid vesicles, niosomes as liposome-related vesicles will also be briefly mentioned in this review. These vesicles are composed of synthetic single-chain or double-chain surfactants (non-ionic, anionic, or cationic), which can be used alone or in combination with cholesterol to form the vesicles [46].

As this review mainly focuses on the role of vesicles in the treatment of acne, being a pilosebaceous-associated skin condition, we will delve further into the ability of liposomes to target skin appendages.

The significance of targeting skin appendages in drug delivery, particularly the pilosebaceous route, has been acknowledged due to its substantial contribution to both topical and transdermal drug administration [47,48]. Researchers from various groups have extensively investigated the potential of liposomes to target the pilosebaceous unit, encompassing the hair follicle, hair shaft, and sebaceous gland [48,49]. Targeting the pilosebaceous unit holds promise in the treatment of conditions associated with hair follicles, such as acne, alopecia, and certain cancers, as well as applications in vaccination, gene therapy, and accelerated systemic drug delivery via the shunt pathway [50]. Notably, liposomes and other nanocarriers have been demonstrated to achieve a higher drug deposition in the hair follicle compared to aqueous, aqueous/ethanolic, and other solutions [47,48,51]. Soya PC vesicles with dimiristoil-PC (DMPC) or deoxycholic acid (DA) enhanced the follicular drug uptake of chloramphenicol by 1.5- and 2-fold, respectively [52]. Liposomal formulations combining dihomo-γ-linolenic acid, S-equol, and propionyl-l-carnitine effectively reduced androgenic-alopecia-related hair loss in both men and women, highlighting liposomes’ potential for hair follicle targeting [53]. Liposomes have also demonstrated the ability to deliver anti-acne drugs to hair follicles, such as adapalene [54], 5-aminolevulinic acid (5-ALA) for PDT, and others, as elaborated in the following sections. Due to the aforementioned advantages, liposomes are expected to be promising nanocarriers for anti-acne drugs, which is going to be discussed in the following sections.

Besides liposomes, other nanocarriers have also been used for dermal drug delivery, including polymeric and solid lipid nanoparticles (SLN), nanostructured lipid carriers (NLC), nanoemulsions, nanosuspensions, polymeric micelles, etc. [55,56,57,58].

**Figure 3:**
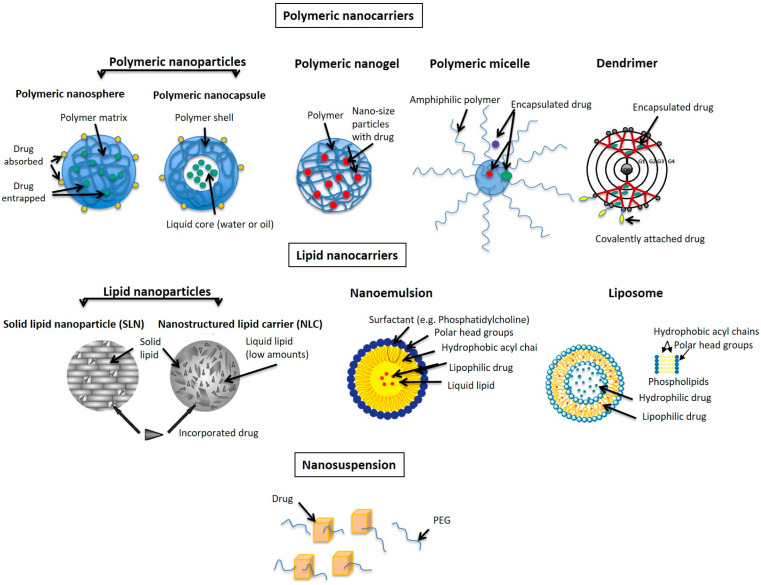
Schematic representation of different nanocarriers (modified from [55]).

Nanoparticles can be classified into lipid-based and polymer-based ones (Figure 3). The first generation of lipid-based nanoparticles comprises solid lipid nanoparticles (SLNs), derived solely from solid lipids like glycerides, waxes, or their combinations (Figure 3). SLNS are characterized by an almost perfect solid lipid matrix, which, during storage, as the lipid molecules tend to increase their order, can lead to the expulsion of the drug from the matrix. Subsequently, the improved second generation, termed nanostructured lipid carriers (NLCs), emerged by blending solid lipids with longer-chain fatty acids and oils containing shorter-chain fatty acids. These lipid nanoparticles have an imperfect lipid matrix consisting of solid and liquid lipids and represent more stable systems. Both kinds of lipid nanoparticles have great potential for dermal drug delivery [56].

Depending on the manufacturing process, polymeric nanoparticles can either be nanospheres (matrix-type nanoparticles) or nanocapsules (reservoir-type nanoparticles) (Figure 3). In addition to non-biodegradable polymers, various biodegradable polymers—both synthetic and natural—have garnered attention and are utilized in their production. Common biodegradable polyester polymers include polylactides and polyglycolides. However, other polymers like hyaluronic acid, chitosan, etc., are also employed (for further details, refer to [59]). It is characteristic of nanoparticles that they mostly remain at the skin surface; however, they are also able to accumulate into hair follicles, and a size dependence could be observed. Namely, the optimal size found for polymeric poly(d,l-lactic-co-glycolic acid) (PLGA) nanoparticles to be able to penetrate deeply into the hair follicles was 300–700 nm (optimal was 320 nm), while larger or smaller particles reached significantly lower penetration depths [60]. This is an important finding indicating that nanoparticles could deliver anti-acne drugs into the hair follicles.

Polymeric micelles are self-assembled colloidal structures formed by amphiphilic block copolymers in aqueous solutions. They consist of a hydrophobic core and a poly(ethylene glycol) (PEG) hydrophilic shell (Figure 3). Thus, the hydrophobic core provides a solubilizing environment for poorly water-soluble drugs or other hydrophobic molecules, while less lipophilic drugs can be located partly in the core and partly in the shell of the micelle, or only in the hydrophilic shell as in the case of hydrophilic drugs. Due to their structure, polymeric micelles have great potential for dermal drug delivery [58,61].

Nanoemulsions have become widely utilized in the enhancement of dermal drug delivery. This term denotes conventional emulsions featuring droplet sizes within the nano range, as illustrated in Figure 3. It is pertinent to note that classical nanoemulsions, stabilized typically by lecithin-type surfactants and produced via high-energy emulsification, seldom achieve mean droplet sizes below 100 nm, despite the established criterion specifying the “nano” prefix for systems ranging between 1 and 100 nm. Nanoemulsions have several advantages over traditional emulsions, including improved physical stability during storage and increased efficacy in dermal drug delivery. Furthermore, their stabilization by skin-friendly surfactants such as lecithins eliminates the need for synthetic surfactants. The versatility of nanoemulsions extends to the incorporation of both hydrophilic and lipophilic actives, rendering them promising vehicles for use in dermatological and cosmetic formulations [62].

One particularly intriguing strategy in recent years for enhancing dermal drug delivery involves the utilization of nanocrystals (Figure 3). The term “nanocrystals” denotes nanoparticles composed solely of a drug substance, devoid of any matrix material, with an average diameter typically below 1 µm (usually ranging between 200 and 500 nm). These nanocrystals are formulated as colloidal suspensions, also known as nanosuspensions (NS), in both aqueous and non-aqueous media. These nanosuspensions are stabilized using either surfactants (ionic or non-ionic) or polymers polymers (such as polyethylene glycols (PEG)) [56,63]. The penetration-enhancing ability of nanosuspensions is attributed to three primary factors: (1) an increase in the drug’s saturation solubility, thereby augmenting the concentration gradient between the formulation and the skin surface; (2) accelerated dissolution kinetics, owing to the amplified particle surface area; and (3) enhanced adherence of nanocrystals to the skin [64].

Dendrimers are hyperbranched polymers characterized by highly controlled, tree-like structures with multiple branches emanating from a central core (Figure 3). Dendrimers have attracted significant attention in various fields, including drug delivery, due to their unique properties, such as a uniform size, well-defined architecture, and the ability to encapsulate drugs or other molecules. Dendrimers have hydrophobic and hydrophilic exteriors, similar to lipid bilayer membranes, enabling them to encapsulate different drugs [65]. We should distinguish between poly-(amidoamine) (PAMAM) dendrimers, showing their drawback of leading to biodegradation products—“acrylates”, and the peptide dendrimers, which show a lower toxicity, as they degrade to harmless amino acids. Both kinds of dendrimers have been used for dermal/transdermal drug delivery [55].

## 4. Topical Retinoids

Topical retinoids have a central role in the treatment of acne. They show comedolytic and anti-inflammatory activities, enhance dyspigmentation, and help to maintain clear skin [3]. Each of them has the ability to bind to a different set of retinoic acid receptors, and, thus, exert different activity, and therapeutic efficacy, as well as skin tolerability [3]. Therefore, regardless of being used alone or combined with other drugs, such as antibiotics or benzoyl peroxide, retinoids have become the mainstay of acne treatment. The European Medicines Agency (EMA) has approved peroral and topical/dermal “retinoid-containing medicinal products“, which may contain one of the following retinoids: aciretin, adapalene, alitretinoin, bexarotene, isotretinoin, tretinoin, tretinoin, or tazarotene, alone or combined with antibiotics (erythromycin or clindamycine) or benzoyl peroxide (please see the following for different products: EMA/PRAC/461930/2016 Rev 1 [66]). In 2018, EMA completed its review of medicines containing retinoids, reporting that measures for pregnancy prevention are necessary [67]. As to topical retinoids, EMA approved adapalene, tretinoin, isotretinoin, alitretinoin, and tazarotene. The FDA approved four topical retinoids for the treatment of acne: tretinoin, adapalene, tazarotene, and trifarotene. The AAD Guideline strongly recommends the use of the aforementioned FDA-approved topical retinoids in mild and moderate to severe acne based on moderate certainty evidence (moderately confident in the effect estimate) obtained in 20 clinical studies. Furthermore, this guideline reported that the obtained results indicate that there is no clear superiority of one topical retinoid over another. Instead, the efficacy and tolerability vary based on specific concentrations and formulations [3]. According to the recommendations of the European evidence-based (S3) guideline for the treatment of acne, adapalene is the preferred choice over tretinoin and isotretinoin [68], while NICE recommends both adapalene and tretinoin [5]. Multimodal topical therapy is always preferred, thus, fixed-dose combinations of topical retinoids with benzoyl peroxide or antiobiotics should be used [3]. However, besides findings that topical retinoids are generally safe and efficacious for the treatment of acne, they have also shown common adverse effects, such as erythema dryness, burning sensation, exfoliation, peeling, and pain [3,69]. The use of topical retinoids may lead to increased sensitivity to sunlight. Consistent daily application of sunscreen alongside retinoid use can help minimize the risk of sunburn [3]. Further, they are lipophilic and, hence, difficult to formulate into preparations, and they are photolabile. In order to increase their skin tolerability and, thus, patient compliance, as well as to circumvent their lipophility and instability, they have been incorporated into different nanocarriers, such as liposomes and other vesicles [9].

Studies reporting the use of liposomes as carriers for different retinoids are going to be discussed in the following sections, while the most important results from the studies and represented in Table 1.

### 4.1. Tretinoin

Topical tretinoin is the most frequently used retinoid for the treatment of acne [9]. NICE recommends a fixed combination of topical tretinoin with topical clindamycin for any acne severity [5]. It represents an anti-acne agent especially for reducing the size and number of comedones. However, like all retinoids, tretinoin shows disadvantages, such as very low water solubility, skin irritation, and high instability in the presence of air, light, and heat, which limits its formulation into preparations, as well as its topical application. The incorporation of tretinoin into different vesicles (e.g., liposomes, niosomes, etc.) circumvents these problems as shown by several researchers [15,70,71,72,73,74]. Tretinoin is also used in the treatment of other proliferative and inflammatory skin diseases, such as psoriasis, eczema, and epithelial skin cancer, and in the treatment of photoaged skin.

#### 4.1.1. Tretinoin-Loaded Conventional and Elastic Liposomes

Investigating the influence of different liposome parameters (composition, size, lamellarity, and charge) on the (trans)dermal delivery of tretinoin and comparing them to the hydroalcoholic solution, oil solution, and marketed Retin-A^®^ cream, it has been shown that negatively charged liposomes significantly improved the hydration of newborn pig skin, as well as the skin accumulation of tretinoin [71] (Table 1). However, there was no evidence found that intact vesicles could penetrate through the skin.

Tretinoin-loaded liposomes and ethosomes, as well as other tretinoin-loaded nanocarriers, such as SLNs and NLCs, which were incorporated into a carbomer gel, were compared to a commercial gel (0.05% tretinoin) (Table 1). All nanocarriers were found to be more biocompatible and effective (regarding psoriasis) than the marketed product in vivo in mice. Nanoparticulate carriers (SLNs and NLCs) offered significantly enhanced photostability, skin transport, and anti-psoriatic activity compared to the vesicular carriers (liposomes and ethosomes) and the marketed product. The following effectiveness of nanocarriers is expected for deep skin disorders (i.e., acne)—SLN = NLC = ethosomes ≫ liposomes—and for superficial skin disorders (i.e., psoriasis)—liposomes = SLN = NLC ≫ ethosomes [15].

Further, ultradeformable liposomes loaded with tretinoin showed, in vitro in pig ear skin, sustained and controlled drug release. Tretinoin was predominantly deposited in the stratum corneum, indicating the topical delivery of tretinoin, which was desired (Table 1). Due to the importance of developing safe, nonirritating topical delivery systems, the cytotoxicity potential of tretinoin–loaded liposomes was determined by Trypan Blue assay, and it was shown that liposomes were not cytotoxic at a 0.05% tretinoin concentration. As to skin irritation, liposomes induced in vivo in mice significantly lower the erythema score compared to the marketed Ketrel^®^ cream containing 0.05% tretinoin. Thus, tretinoin–loaded ultradeformable liposomes could be considered as a promising dermal delivery system for tretinoin without inducing skin irritation (unlike other commercial formulations), which is advantageous for the treatment of skin diseases, such as acne, etc. [37].

**Table 1:** Vesicles used as carriers for topical retinoids.

Formulation	Drug	Therapeutic Indication	Experimental Condition	Outcome	Reference
**Retinoids** **Tretinoin-loaded liposomes**					
Positively or negatively charged MLV or UV *: hydrogenated soya PC ** (Phospholipon 90H) or non-hydrogenated soya PC (Phospholipon90) and cholesterol (chol), with stearylamine (SA) or dicetylphosphate (DCP)	Tretinoin	Acne vulgaris	In vitro, newborn pig skin	Negatively charged liposomes significantly enhanced the tretinoin penetration into the skin and its skin accumulation compared to the hydroalcoholic solution, oil solution, and marketed Retin-A^®^ cream. The skin hydration was also increased.	[71]
Ultradeformable liposomes (containing phosphatydylcholine (PC) and Polysorbate (Tween^®^ 80)	Tretinoin	Acne vulgaris	In vitro, pig ear skin	Tretinoin was predominantly deposited in the SC, less in the epidermis and dermis with no drug detected in receptor compartment over 24 h, indicating only topical delivery of tretinoin. Liposomes were not cytotoxic at 0.05% tretinoin concentration.	[37]
			In vivo, mice	Liposomes induced lower erythema score than the Ketrel^®^ cream containing 0.05% tretinoin.	
Liposomes (PC, CHOL, normal saline), ethosomes (PC, ethanol, normal saline), solid lipid nanoparticles, (SLN; PC, Compritol 888, Tween 80, ethanol, water), nanostructured lipid carriers (NLC; PC, Compritol 888, Tween 80, Isopropyl myristate, ethanol, water) were incorporated into a carbomer (1,5% Carbopol 934) gel, and compared to a commercial gel (same tretinoin concentration, 0.05%)	Tretinoin	Psoriasis Prediction of acne treatment	In vivo, mice (antipsoriatic activity in mouse tail model)	All nanocarriers were more biocompatible and effective in treating psoriasis than the marketed product. Liposomal gel showed the highest skin retention (% of dose applied) of tretinoin compared to other nanocarriers and the marketed cream: Liposomal gel 8.12 ± 0.09%; Ethosomal gel 3.31 ± 0.11%; SLN gel 4.28 ± 0.08%; NLC gel 5.62 ± 0.12%; Commercial gel 1.52 ± 0.04%. Liposomes, SLN, NLC showed good tolerability; ethosomal gel showed some inflammation, while the marketed cream showed highest inflammation (and lowest skin retention, permeation, and effectiveness).	[15]
**Tretinoin-loaded liposomes in clinical studies**					
Liposomes (tretinoin, 0.01%) were compared to commercial gels (either 0.025% or 0.05% tretinoin) Patients received, once daily for 10 weeks, on one side the liposomal tretinoin, and the commercial gels on the other body side.	Tretinoin	Acne vulgaris	In vivo, double-blind clinical study, 20 patients with uncomplicated acne vulgaris.	Liposomal tretinoin was therapeutically more effective. As rated by the patients, liposome-encapsulated tretinoin induced less burning than the 0.025% gel and the 0.05% gel and less erythema than the 0.025% gel. Further, liposomal tretinoin was also better tolerated as to the rating by the investigator.	[75]
Liposomes were incorporated into a carbomer gel, and compared to a comercial tretinoin gel.	Tretinoin	Acne vulgaris	In vivo, double-blind clinical study, 30 patients with uncomplicated acne vulgaris. 3-month treatment duration.	Tretinoin-liposomal gel provided a 1.5-fold enhancement of drug efficacy compared to a marketed conventional gel. Highest improvement was observed in the treatment of comedones, where the mean percent reduction in lesions increased from 62.36% for a conventional tretinoin gel to 94.17% for a liposomal tretinoin gel. Liposomal tretinoin gel decreased all side effects (erythema and irritation) compared to the conventional tretinoin gel.	[76]
Different liposomes were developed; PC–chol–DCP (9:1:0.01) liposomes (0.025% tretinoin) were incorporated into a 1% carbomer gel.	Tretinoin	Acne vulgaris	In vivo, double-blind clinical study, 4 weeks treatment duration.	Liposomal gel showed a significantly superior efficacy compared to the marketed product. Liposomal tretinoin gel provoked a significantly lower erythema score compared to the marketed tretinoin gel, thereby showing improved skin tolerability and patient compliance.	[77]
**Tretinoin-loaded penetration-enhancer-containing vesicles (PEVs)**					
PC-vesicles (control liposomes) with added different hydrophilic penetration enhancers: decylpolyglucoside (Oramix^®^ NS10, OrNS10), caprylocaproyl macrogol 8-glyceride (Labrasol^®^, Lab), 2-(2-ethoxyethoxy)ethanol (Transcutol^®^ P, Trc), and propylene glycol (PG).	Tretinoin	Acne vulgaris	Ex vivo, newborn pig skin	Improved cutaneous drug accumulation and a reduced transdermal delivery of tretinoin with all PEVs, except with PG-PEVs. Higher skin accumulation with PEVs containing only encapsulated tretinoin (dyalized vesicles) compared to vesicles containing the drug inside and outside the vesicles (non-dyalized). Skin accumulation increased in the order: control PC-liposomes < PG-PEVs < Trc-PEVs ≤ Or-PEVs < Lab-PEVs. Dialysed PEVs provided the highest drug accumulation in SC and epidermis, while dialyzed Or-PEVs delivered similar high drug amounts to all skin layers, including the dermis. Conventional liposomes showed a good LAC, while, among PEVs, only Or-PEVs showed higher LAC, because Lab- and Trc-PEVs provided not only a high drug deposition in the skin, but also an enhanced flux that reduced LAC.	[78]
Liposomes (Phospholipon^®^ 50 (PC)), PEVs (composed of PC and Labrasol^®^ (1.2:1)), niosomes (diolein (Plurol^®^ Oleique CC), cholesterol (5:1)), niosomes (diolein, Labrasol^®^ (1:1)) 0.25 mg/mL tretinoin	Tretinoin	Acne vulgaris	Ex vivo, newborn pig skin	All vesicles enhanced drug accumulation in the skin with no drug permeation. Conventional liposomes showed the highest drug deposition in the total skin (18% of the applied dose), followed by Lab-niosomes (13%), Lab-PEVs (9%), and diolein-niosomes (7.0%). The highest drug deposition was always found in SC, and this was important when liposomes and Lab-niosomes were used (15% and 11%, respectively), while diolein-niosomes showed the highest drug delivery in the dermis, and the Lab-PEVs (2%) in the epidermis.	[79]
Different PEVs (containing soy PC and Transcutol^®^ or Oramix^®^ NS10 or Labrasol^®^ or PG) and tretinoin 0.05% cream	Tretinoin	Acne vulgaris	In vitro, mice skin	Optimized PEVs provided higher drug penetration into the skin than the tretinoin 0.05% cream, while LAC was highest for the tretinoin cream. PEVs decreased erythema, peeling, and burning.	[80]
**Isotretinoin-loaded liposomes**					
Small unilamellar vesicles (SUV), multilamellar vesicles (MLV), preformed concentrated vesicles (Natipide^®^ II, Lipoid, Ludwigshafen am Rhein, Germany), mixed micelles of lecithin and bile salt.	Isotretinoin	Acne vulgaris	In vitro	No significant difference between ethanolic or liposomal or micellar gel; Natipide^®^II formulation resulted in significantly lower isotretinoin concentrations in sebaceous glands compared to the ethanolic gel. Isotretinoin concentration in the sebaceous glands varied between 0.17 and 1.57 ng/mg tissue. Drug penetration into sebaceous glands was along the follicular route.	[81]
Hydroxypropyl β cyclodextrin (HP-β-CD)-drug complex, encapsulated into elastic liposomes	Isotretinoin	Acne vulgaris	In vitro, skin permeation and deposition study	The transdermal drug flux for different vesicles was from 10.5 ± 0.5 to 13.9 ± 1.6 μg/cm^2^/h, being approx. 15–21-folds higher than that of the drug solution (0.7 ± 0.1 μg/cm^2^/h) and 4–5-folds higher than that of the drug-CD complex in solution (2.7 ± 0.1 μg/cm^2^/h). The skin deposition of the drug increased significantly by cyclodextrin complexation (30.1 ± 0.1 μg), and its encapsulation into elastic liposomes further increased its skin deposition (262.2 ± 21 μg).	[82]
			In vivo, Draize test	The skin irritation study also showed that isotretinoin inclusion complex in elastic liposomes significantly reduced skin irritation compared to free drug.	
**Adapalene-loaded liposomes**					
Liposomes (72% Phospholipon 90H^®^ and 28% cholesterol) with 1% adapalene Differin^®^ gel (1% adapalene) 1% adapalene solution in PEG 400	Adapalene	Acne vulgaris	In vitro, porcine ear skin	Liposomes delivered higher drug amounts (µg/cm^2^) into hair follicles than conventional Differin^®^ gel (1% than other formulations: Liposomes: 6.72 ± 0.83; Differin^®^ gel: 3.33 ± 0.26; Adapalene solution: 1.62 ± 0.054. Liposomes significantly increased the skin deposition of tretinoin compared to other two formulations: Liposomes: 1.75 ± 0.33; Differin^®^ gel: negligible; Adapalene solution: 0.92 ± 0.26. Confocal microscopy confirmed drug penetration into hair follicles by using liposomes.	[54]
Different transfersomes (PC:Chol:Tween^®^ 80:sodium deoxycholate); amount of their components was varied; optimized transfersomes were incorporated into a 1% carbomer gel (containing also dissolved vitamin C); Three gels (1% adapalene) were compared: transfersomal gel with vitamin C, transfersomal gel without vitamin C, and Adiff aqueous gel.	Adapalene	Acne vulgaris	In vivo, irritation study, rats (Draize method)	The optimized transfersomal gel showed no signs of irritation over 72 h, in contrast to the Adiff aqueous gel (1% adapalene), which exhibited mild irritation and moderate erythema.	[83]
			In vivo, rats with testosterone-induced acne	Transfersomal gel with vitamin C demonstrated a significant reduction in inflammatory lesions (papules, pustules, cysts) and total lesion counts. Acne lesions improved by over 50% with the transfersomal gel and over 65% with the vitamin-C-containing transfersomal gel in just 4 weeks. Papule counts were 24, 17, and 14 in groups treated with the marketed gel, transfersomal gel, and transfersomal gel containing vitamin C, respectively, compared to 50 papules in the group treated only with testosterone. For comedones, approximately 50%, 75%, and 95% disappeared with the marketed gel, transfersomal gel, and transfersomal gel containing vitamin C, respectively. Histopathology images revealed the disappearance of swollen sebaceous glands in rats treated with the optimized adapalene-loaded transfersomal gel with or without vitamin C. Side effects like erythema, scaling, and dryness were observed in rats treated with the marketed gel, partially seen in rats treated with adapalene-loaded transfersomal gel, but not in rats treated with the transfersomal gel containing vitamin C.	
Adapalene- and benzoyl-peroxide-loaded liposomes were incorporated into a carbomer gel; Compared to Epiduo^®^ (0.1% adapalene: 2.5% benzoyl peroxide, Galderma Laboratories LP, Fort Worth, TX, USA).	Adapalene and Benzoyl peroxide	Acne vulgaris	Ex vivo dermal bioavailability, dermal distribution	Liposomal gel significantly enhanced dermal bioavailability of drugs: Adapalene—2.1, 5.4-fold; Benzoyl peroxide—3.0, 7.83-fold, as compared with free drugs and Epiduo^®^, respectively.	[84]
			In vivo anti-acne efficacy and skin irritation studies, animal model	Liposomal gel reduced papule density and skin irritation as compared with free drugs and Epiduo^®^.	
**Tazarotene-loaded niosomes**					
Niosomes (Tween^®^ 20: cholesterol) and nansponges (ethyl cellulose, PVA) were incorporated into carbomer 940 gel. Gels were compared to niosomes, nanosponge, plain gel, and marketed Tazorac^®^ formulation.	Tazarotene	Acne vulgaris	In vitro permeation, rat skin	Niosomal and nanosponge gels showed lower skin permeation of the drug, but a 2–3-times-higher skin deposition of the drug compared to Tazorac and other formulations.	[85]

* MLV—multilamellar vesicles, SUV—small unilamellar vesicles, UV—unilamellar vesicles; ** LAC (local accumulated concentration) = drug accumulated in the skin/drug permeated through the skin.

As to clinical studies, there are only few of them investigating the therapeutic efficacy of tretinoin-loaded vesicles in patients suffering from acne (Table 1). One of the first clinical studies was performed already in the 1990s [75], at a time when the advantages of liposomal over conventional topical preparations have been discovered. The efficacy and local tolerability of liposomal tretinoin 0.01% were compared to those of a commercial gel with either 0.025% or 0.05% tretinoin. Liposomal tretinoin appeared to be potent, i.e., therapeutically effective, compared to the reference gels, however, inducing only a slightly more rapid clearing of comedones. Regarding skin irritancy, liposomal tretinoin was superior. Thus, liposomes with tretinoin allow the reduction of the concentration of tretinoin without decreasing its efficacy, and increase its skin tolerability. These findings encouraged the development of different liposomes and other vesicles as carriers for tretinoin to enhance its skin penetration, skin tolerability, and therapeutic effectiveness. Furthemore, the superiority of a liposomal tretinoin gel over a conventional marketed tretinoin gel, regarding enhanced therapeutic effectiveness and skin tolerability, was reported also by Vandana et al. [76]. Namely, the liposomal tretinoin gel decreased all side effects associated with tretinoin therapy, such as erythema and irritation, compared to the conventional tretinoin gel. In addition, tretinoin (0.025%)-loaded liposomes composed of PC–cholesterol–dicetylphosphate (9:1:0.01) and incorporated into a 1% carbomer gel showed significantly superior therapeutic efficacy over 1, 2, and 3 weeks, as well as improved skin tolerability and patient compliance compared to the marketed product over a 4-week period [77].

#### 4.1.2. Tretinoin-Loaded Penetration-Enhancer-Containing Vesicles (PEVs)

In order to improve the dermal delivery of tretinoin, Manconi et al. developed a new generation of vesicles, i.e., the penetration-enhancer-containing vesicles (PEVs), which, besides PC, also contain different hydrophilic penetration enhancers (Table 1) [78]. Ex vivo permeation experiments showed an improved cutaneous drug accumulation and a reduced transdermal delivery of tretinoin with PEVs, except for propylene glycol (PG)-PEVs. The results revealed that decylpolyglucoside (Or)-PEVs were the best carriers for the cutaneous delivery of tretinoin, i.e., better than conventional liposomes and other PEVs. It has also been shown that PEVs are able to strongly interact with intercellular lipids causing an enlargement of this region [78].

Manca et al. [79] compared different tretinoin-loaded vesicles, i.e., liposomes, PEVs, and niosomes (Table 1). Conventional liposomes showed the highest drug deposition in the total skin. The highest drug deposition ex vivo in newborn pig skin value was always found in the SC, and this was particularly important when conventional liposomes and Labrasol^®^(Lab)-niosomes were used, while diolein-niosomes showed the highest drug delivery in the dermis, and the Lab-PEVs in the epidermis. Diolein–niosomes were less efficient carriers for tretinoin than liposomes, while a Labrasol^®^ addition improved the skin deposition of tretinoin only when it was added to niosomes. Thus, according to the authors [79] and previous results [78], Labrasol^®^ does not simply act as a penetration enhancer, but it also influences vesicle properties, thereby influencing their interaction with the skin. Confocal laser microscopy (CLSM) studies showed for all vesicles a deposition of both hydrophilic and lipophilic probes in the superficial SC. An important finding was the observed deposition of both fluorescent labels from Lab-niosomes in skin appendages, suggesting that Lab-niosomes are a promissing delivery system for tretinoin in the treatment of dermatological disorders associated with hair follicles and sebaceous glands (such as acne, alopecia, and several skin tumors) [79].

Bavarsad et al. developed different PEVs containing soy PC and Transcutol^®^ for the topical delivery of tretinoin [81]. The results revealed a higher in vitro drug penetration through mice skin for the optimized PEVs than for the tretinoin 0.05% cream, which could be due to the solubilizing properties of Transcutol^®^ (Table 1). Histological studies in mice revealed that optimized PEVs compared to the tretinoin cream caused milder hyperkeratosis without hyperplasia. The authors suggested that the deformable PEVs could gradually release tretinoin and, thus, decrease its adverse effects, such as erythema, peeling, and burning, and also increase patient compliance.

#### 4.1.3. Tretinoin-Loaded Niosomes

The main aim of this review is to represent different liposomes. However, we will, in brief, also discuss niosomes as they are structurally similar to liposomes, and have been most often studied together with liposomes.

Niosomes loaded with tretinoin and bicalutamide (BCT) were developed [51]. Bicalutamide (BCT) represents a non-steroidal anti-androgenic agent, being lipophilic and possessing a log P of 2.3. These niosomes were designed for targeting the hair follicles and pilosebaceous units. The Design-Expert software v12 was employed for formulation optimization as the formulations contained different ingredients (Precirol, stearic acid, Capryol PGMC, triolein, cholesterol, and surfactatnt mixture). In vivo, in male golden hamsters, follicular targeting was assessed using rhodamine B-loaded niosomes to trace skin penetration pathways (Table 1). Animal study results indicated a significant accumulation of the prepared niosomes, with an average diameter of about 300 nm, in hair follicles, which confirmed the finding of Manca et al. [80], that niosomes could be used for follicular targeting.

#### 4.1.4. Tretinoin-Loaded Microsponge Gels, Solid Lipid Nanoparticles, Nanostructured Lipid Carriers, Nanosuspension, and Nanoemulsion

Tretinoin was encapsulated within porous microspheres made of methyl methacrylate/glycol dimethacrylate copolymer, with diameters ranging from approximately 100 to 250 nm (Microsponge Delivery System (MDS)), which were further suspended into a carbomer-based gel. This microsphere gel was launched on the market as the Retin-A Micro^®^ gel (Valeant Pharmaceuticals International, Inc., Laval, QC, Canada), which contains tretinoin in concentrations of 0.04%, 0.06%, 0.08%, and 0.1% [86]. Furthermore, during a 12-week treatment period, the Tretinoin microsphere gel 0.04% demonstrated significant superiority over the vehicle gel in reducing both inflammatory and noninflammatory lesions. Additionally, it achieved a higher successful treatment rate compared to the vehicle gel, as assessed by IGE ratings at week 12. Common adverse events included mild erythema, peeling, and dryness, occurring in 59.7% to 63.3% of patients using TMG 0.04%, compared to 26.9% to 51.0% with the vehicle gel (placebo) [87]. The concept behind this delivery system is that these sponge-like porous microspheres slowly release the drug, reducing irritation and enhancing stability. This MDS has previously been utilized for benzoyl peroxide aimed for acne treatment [88]. However, MDS maintained the tretinoin efficacy, while the skin irritation was still present, indicating the need for new drug delivery systems which would also reduce the skin irritattion.

Tretinoin was encapsulated into solid lipid nanoparticles (SLNs), which significantly enhanced its photostability compared to the methanolic tretinoin solution, and prevented isomerization [72]. Skin irritation studies on rabbits demonstrated that the SLN-based tretinoin gel was significantly less irritating compared to the marketed tretinoin cream, highlighting its potential to improve skin tolerability. In vitro permeation studies in rat skin indicated that the SLN-based tretinoin gel exhibited a permeation profile similar to that of the marketed TRE cream.

SLNs prepared from natural solid lipids were developed as carriers for tretinoin in order to enhance its skin tolerability [89]. Tretinoin-loaded SLNs-based gels were formulated by incorporation of SLNs into a gel vehicle. These gels were evaluated for their potential to induce skin irritation, occlusivity, and skin penetration ability. Compared to a commercial product, the SLNs-gel induced lower skin irritancy, thereby showing a higher skin tolerability, occlusivity, and slower drug release. Thus, the obtained results confirmed the safety of the SLN-based gels due to the favorable composition of SLNs.

In addition, SLNs containing tretinoin, with and without chitosan, were prepared to assess their cytotoxicity in keratinocytes and antibacterial activity against acne-related bacteria [90]. Tretinoin-loaded chitosan-SLNs showed a high encapsulation efficiency, exhibited no cytotoxicity to keratinocytes, and displayed significant antibacterial activity against *C. acnes* and *S. aureus*. Therefore, chitosan-SLNs hold promise for enhancing the therapeutic efficacy of tretinoin in the topical treatment of acne.

Nanostructured lipid carriers (NLCs) containing stearic acid, oleic acid, and Tween^®^ 80 and Span^®^ 60 as a solid lipid, liquid lipid, and surfactants, respectively, were loaded with tretinoin in order to reduce its skin irritation potential, increase its loading capacity, and prolong its duration of action. The loaded NLCs were further incorporated into carbomel gels, which indeed showed a sustained drug release pattern. In addition, the in vivo skin irritation test performed in male Wister rats revealed no irritation or erythema after the application of the NLC-gel in comparison to the marketed tretinoin gel showing irritation and slight erythema (within 3 days) [91].

An NLC drug delivery system containing tretinoin was compared to a conventional 0.05% tretinoin cream in the treatment of mild to moderate acne vulgaris. The therapeutic efficacy was assessed in a double-blind, split-face randomized clinical study, comparing acne lesions, porphyrin production, and skin parameters between sides treated with the NLC-tretinoin formulation and with the conventional tretinoin cream. The decrease in acne lesions was significantly greater on the side treated with the NLC-tretinoin formulation. Moreover, the porphyrin production in the pilosebaceous follicles decreased significantly only when treated with the NLC-tretinoin formulation. Thus, the encapsulation of tretinoin into NLCs led to superior therapeutic efficacy, a high tretinoin-loading capacity in the drug delivery system, and high safety due to low plasma concentrations of the drug [92].

Tretinoin was also encapsulated into an oil-in-water nanoemulsion. The therapeutic efficacy of the tretinoin-loaded nanoemulsion was compared to that of the conventional 0.05% tretinoin emulsion through comparing the number of acne lesions and porphyrin production in both sides of the face. Each side of the face was treated with one of the two investigated formulations [93]. The clinical trial revealed that both the number and size of acne lesions and the intensity of porphyrin production noticeably decreased following the application of the tretinoin-loaded nanoemulsion. Therefore, the nanoemulsion formulation was superior to the conventional emulsion in terms of therapeutic efficacy.

Nasrollahi et al. [94] investigated, in vivo in twenty healthy volunteers, the safety of a tretinoin-loaded nanoemulsion and NLCs by determining changes in human skin biophysical parameters, such as skin hydration, transepidermal water loss (TEWL), erythema, and pH on their volar forearms, before and after applying the lotions. The results showed that both formulations were safe to be applied onto human skin and that there was no significant difference between their effects.

Lai et al. [95] investigated whether a nanosuspension could increase the dermal delivery and photostability of tretinoin. As to the dermal delivery, the nanosuspension localized the drug in the pig skin with minimal transdermal delivery, while the nanoemulsion (control) significantly enhanced drug permeation. Regarding photodegradation studies, the nanosuspension was compared to the nanoemulsion and a methanolic solution, under UV irradiation (1 h, λ = 366 nm). The nanosuspension notably improved tretinoin stability during UV irradiation compared to both controls. These findings suggest that nanosuspension could be a valuable formulation for enhancing tretinoin dermal delivery and stability.

With regard to NLCs and SLNs, compared to vesicles, Raza et al. [15] reported that nanoparticulate carriers demonstrated improved photostability, skin penetration, and anti-psoriatic activity compared to vesicular carriers, such as liposomes and ethosomes, as well as the marketed product. The results were the opposite for the acne therapy, as explained in the previous section about tretinoin-loaded vesicles. Furthermore, all developed nanocarriers exhibited a higher biocompatibility and effectiveness than the marketed product.

### 4.2. Isotretinoin

Isotretinoin was used orally for the treatment of severe acne. However, the oral use of isotretinoin has drawbacks due to the severe side effects such as hypertriglyceridemia, dry skin, ocular side effects, hypercalcemia, and mood disorders. Further, isotretinoin is a highly lipophilic drug with poor oral bioavailability (25%) [96]. Therefore, in order to avoid the disadvantages of its peroral use, isotretinoin was used topically in the form of creams and gels in the treatment of acne vulgaris and other dermatological disorders. However, like in the case of tretinoin, its topical application is limited by several drawbacks, such as skin irritation, very low water solubility (which makes it difficult for the incorporation into formulations), and photodegradation, which renders the drug ineffective and may cause skin allergies due to degradation products. An additional problem is that isotretinoin does not show sufficient clinical efficacy in all acne lesions as it is assumed that only low amounts of isotretinoin (being pharmacologically insufficient) are delivered to the sebaceous glands, representing its target site, which lead to the failure of the desired therapeutic effect. In order to overcome these limitations, isotretinoin has been incorporated into nanocarriers.

#### 4.2.1. Isotretinoin-Loaded Conventional and Elastic Liposomes

One of the first studies investigating liposomal isotretinoin for enhanced follicular delivery was performed already in the 1990s (Table 1) [81]. In a later study performed by Kaur et al. [82], isotretinoin was incorporated into elastic liposomes (transfersomes) using a hydroxypropyl β cyclodextrin (HP-β-CD)-drug complex, offering a unique approach merging the advantages of both carriers. Cyclodextrin complexation, known for improving the solubility and stability of drugs, was combined with elastic liposomes, that had been recognized for enhanced skin delivery and reduced side effects [42]. In vitro studies demonstrated a 15–21-times-higher transdermal drug flux from different vesicles compared to the drug solution, and a 4–5-times-higher effect than the drug-CD complex in solution (Table 1). Cyclodextrin complexation significantly increased isotretinoin skin deposition, and encapsulating this complex into elastic liposomes further enhanced drug’s skin deposition. The isotretinoin inclusion complex in elastic liposomes notably reduced the skin irritation potential compared to the free drug. This dual approach using elastic vesicles and cyclodextrin complexes shows promise for effectively targeting isotretinoin to the skin, providing prolonged drug release, minimized photodegradation, reduced skin irritation, and improved topical drug delivery [82].

#### 4.2.2. Isotretinoin-Loaded Solid Lipid Nanoparticles

Isotretinoin was also encapsulated into SLNs composed of the lipid glycerol distearate (type I) (Precirol^®^ ATO 5) and surfactants Tween^®^ 80 and soybean lecithin as stabilizers of SLNs [97]. Results obtained from permeation studies performed in vitro in rat skin revealed that all SLN formulations prevented systemic absorption of isotretinoin through the skin in contrast to the tincture (control). Furthermore, the tretinoin-SLNs composed of 3.0% Precirol^®^ ATO 5, 4.0% soybean lecithin, and 4.5% Tween^®^ 80 showed adequate stability and significantly enhanced the skin accumulation of tretinoin. These findings indicate that tretinoin-loaded SLNs indeed have the potential for skin targeting.

In a later study, performed in vitro in mice skin, it was confirmed that the encapsulation of isotretinoin into SLNs enhances its accumulation in the different layers of the skin, including stratum corneum, while preventing its systemic absorption [98]. In addition, the results revealed that the entrapment of isotretinoin into SLNs significantly minimized its photodegradation, as well its irritating potential compared to the marketed isotretinoin gel.

A recent study investigated SLNs composed of glyceryl mono-stearate (used as lipid) and Tween^®^ 80: butanol (surfactant mixture) as carriers for isotretinoin and α-tocopherol acetate. The aim of developing these SLNs was to decrease the skin irritation induced by isotretinoin, as well as to increase its anti-acne efficacy of isotretinoin, which was studied in rats [99]. The authors reported that the isotretinoin and an α-tocopherol-acetate-loaded SLN gel exhibited sustained drug release for 24 h, inducing no erythema or edema in rabbits, while showing potent efficacy in rat model of acne. Thus, the SLN gel has a high potential to be used as a non-irritant and efficacious formulation for the treatment of acne vulgaris.

#### 4.2.3. Isotretinoin-Loaded Dendrimers

A self-assembled dendrimer-conjugated system was developed to achieve the enhanced percutaneous penetration of isotretinoin [65]. The authors reported that the isotretinoin-loaded dendrimers showed a remarkable controlled release, characterized by a slow release in normal tissues but accelerated release in tissues with low pH, such as sites of inflammation. The skin penetration was studied in vitro in pig skin, the irritation potential in BALB/c mice, and the anti-acne effect in New Zealand white rabbits. Dendrimers loaded with isotretinoin exhibited indeed a high therapeutic efficacy in acne models, afforded better skin penetration than free isotretinoin, and allowed lesion targeting. Additionally, adapalene-loaded dendrimers induced minimal skin irritation as no erythema or slight erythem was seen in contrast to free isotretinoin inducing moderate-to-severe erythema. Further, the dendrimers modestly stimulated the proliferation of sebaceous gland cells and showed no toxicity towards them. Thus, adapalene-loaded dendrimers represent a safe and effective isotretinoin formulation for treating patients with acne vulgaris, and other skin diseases, such as psoriasis.

### 4.3. Adapalene

Adapalene shows a high therapeutic effectiveness in treating acne [69]. NICE recommends a fixed combination of topical adapalene erythema with topical benzoyl peroxide for any acne severity, while, for moderate to severe acne, NICE also recommends oral lymecycline or oral doxycycline besides this combination of topical drugs [5]. Among topical retinoids, adapalene provides the best skin tolerability, although it also shows side effects, such as dry skin, followed by peeling of the skin, and [69]. In addition, it shows a high lipophilicity and pKa of 4.23, which limit its skin penetration and, thus, its bioavailability in the skin and appendages. These drawbacks could be circumvented by the use of nanocarriers, which would enable its formulation into topical preparations and enhance its skin penetration, as well as its targeted delivery to the pilosebaceous unit, and reduce its side effects, thereby increasing patient compliance. Adapalene has been encapsulated in different nanocarriers, such as solid lipid nanoparticles, microemulsions, and acid-responsive carriers, etc. [100,101,102].

#### 4.3.1. Adapalene-Loaded Conventional and Elastic Liposomes

Adapalene-loaded liposomes delivered a 2-fold-higher drug amount to the hair follicles in vitro in pig ear skin than the gel and a 4-fold-higher amount than the drug solution (Table 1). The same trend was seen regarding drug accumulation in the skin layers (epidermis and dermis). CLSM images confirmed that the liposomal formulation delivered the drug into the hair follicles, which is the desired target site in the treatment of acne [54].

In a recent study, different adapalene-loaded transfersomes (PC:Chol:Tween^®^ 80:sodium deoxycholate) were developed. The optimized transfersomes were incorporated into a 1% carbomer gel [86]. The addition of Vitamin C to the formulation aimed to enhance the treatment’s effectiveness, leveraging its antioxidant properties, collagen synthesis promotion, and depigmentation activity [103]. Additionally, it was incorporated to mitigate the side effects typically associated with conventional monotherapy using adapalene. Since vitamin C is unstable in formulations when exposed to air due to its oxidation, ascorbyl-6-palmitate, with a higher stability, was used. Furthermore, the ascorbyl-6-palmitate within the transfersomal gel exhibits strong antioxidant activity, thereby providing effective skin protection and promoting healing in skin diseases. The optimized transfersomal gel exhibited no irritation symptoms (Table 1). Further, the three different adapalene gels (transfersomal gel with or without vitamin C, and Adiff^®^ gel) showed a significant reduction in the overall acne papules. The transfersomal gel with vitamin C significantly reduced the inflammatory lesions and total lesion counts, as well as the comedones, thereby showing highest therapeutic effectiveness (Table 1). Regarding side effects, they were not seen in rats treated with the transfersomal gel containing vitamin C, in contrast to other formulations. The obtained results showed a synergistic effect of adapalene and vitamin C, suggesting that this transfersomal gel is promising in the treatment of acne due to providing enhanced therapeutic efficacy, while excluding adapalene side effects (redness and inflammation), compared to the marketed product and the transfersomal gel without vitamin C [83].

Recently, an adapalene-loaded liposomal formulation was prepared by a novel spontaneous phase transition method (SPT) [104]. The optimized lipoosmes were incorporated into a carbomer gel. In vitro release studies showed a sustained drug release of 79 ± 0.02% of the drug within 24 h. Ex vivo permeability studies demonstrated that 43 ± 0.06 µg/cm^2^ of the drug permeated through the skin within 24 h, with only 28.27 ± 0.04% retained on the epidermis. In vivo studies using a testosterone-induced acne model in mice showed a significant improvement in acne lesions, with no visible scars or inflammation. Thus, the adapalene-loaded liposomal gel holds promise as a safe and effective carrier system for adapalene delivery.

#### 4.3.2. Co-Delivery of Adapalene-Loaded and Benzoyl-Peroxide-Loaded Liposomes

Adapalene has often been used together with benzoyl peroxide in conventional preparations for the treatment of acne vulgaris, as shown by different clinical studies. [105,106,107]. Furthermore, NICE recommends this fixed combination of drugs for the treatment of acne [5]. The encapsulation of these drugs into liposomes has also been studied in order to further enhance their efficacy and safety. Thus, a carbomer hydrogel containing benzoyl-peroxide- and adapalene-loaded liposomes was investigated for its therapeutic efficacy in the treatment of acne, as well as for its tolerability. Ex vivo dermal bioavailability, dermal distribution, in vivo anti-acne efficacy, and skin irritation studies were conducted in an animal model, with comparisons made to the marketed formulation Epiduo^®^ (0.1% adapalene: 2.5% benzoyl peroxide, Galderma Laboratories LP, Fort Worth, TX, USA). The results indicated that the liposomal gel significantly increased the dermal bioavailability of adapalene and benzoyl peroxide, leading to reduced papule density and skin irritation compared to free drugs and Epiduo^®^ (Table 1). These findings affirm that the liposomal gel serves as a therapeutically effective and safe approach for co-delivering the two anti-acne drugs, adapalene and benzoyl peroxide [84].

#### 4.3.3. Adapalene-Loaded Solid Lipid Nanoparticles

SLNs represent an efficacious alternative to liposomes. Thus, adapalene was incorporated into SLNs, which were further formulated into a carbomer hydrogel [108]. The adapalene-loaded SLNs gel exhibited a promising potential in targeting the skin’s epidermal layer and reducing systemic penetration. This system effectively prevented the systemic uptake of adapalene through the skin, localizing the drug in the skin epidermis, as confirmed in a rat skin model. Our findings underscore the potential of SLNs as a nanocarrier for the topical delivery of adapalene in anti-acne therapeutic approaches.

In order to increase the skin accumulation of adapalene, as well as to decrease photosensitization and skin irritation, adapalene was incorporated into SLNs, which were further formulated into a gel [109]. The gel containing adapalene-loaded SLNs exhibited, in vitro in human cadaver skin, a 2-fold increase in the skin accumulation of adapalene compared to the conventional adapalene gel. Furthermore, skin irritation assessments confirmed the safety of the adapalene-loaded SLN gel upon topical application. Consequently, it was inferred that the investigated adapalene-loaded SLN gel, with its skin-localizing ability, holds promise as a carrier for the topical delivery of adapalene.

To mitigate skin irritation, adapalene was encapsulated within SLNs utilizing the ion pair strategy. These SLNs were then incorporated into a hydrophilic gel formulation [110]. Comparative analysis revealed that the adapalene-loaded gel exhibited sustained drug release, epidermal targeting as shown ex vivo in pig ear skin, and reduced skin irritation in hairless mice compared to the commercially available adapalene gel (Differin^®^). These findings confirmed the superiority of the SLN gel compared to Differin^®^ gel, thereby underscoring the efficacy of encapsulating adapalene into SLNs via the ion pair strategy, being a valuable approach to alleviate adverse skin reactions associated with adapalene, potentially enhancing patient adherence to treatment.

Adapalene was encapsulated into a tea tree oil nanoemulsion, which was further incorporated into a carbomer gel [111]. The therapeutic effectiveness and safety of the 0.1% adapalene-loaded nanoemulsion gel were evaluated in comparison to a commercially available gel containing 0.1% adapalene. Both formulations revealed the negligible in vitro permeation of adapalene through the skin, and no drug was found in the liver and plasma in rats after 90 days of treatment. The adapalene-loaded nanoemulsion gel led to a higher retention of adapalene in the dermis compared to the marketed gel. As to the antibacterial activity against *C. Acnes*, the adapalene-loaded nanoemulsion gel effectively reduced the minimum inhibitory concentration compared to a tea tree oil nanoemulsion and pure tea tree oil. In vivo skin irritation studies revealed no irritancy associated with the adapalene-loaded nanoemulsion gel. Thus, this adapalene-loaded tea tree nanoemulsion gel could be used as a novel carrier for the topical delivery of adapalene.

#### 4.3.4. Adapalene-Loaded Polymeric Nanoparticles, Polymeric Micelles, and Dendrimers

In order to develop a formulation which would decrease the irritation potential of adapalene, while achieving follicular drug delivery, adapalene was effectively encapsulated into poly-ε-caprolactone nanospheres (NSs) [112]. These adapalene-loaded NSs were then incorporated into either hydroxypropyl methylcellulose (HPMC) or hyaluronate (HA) gel matrices. The adapalene-loaded NSs revealed ex vivo a significantly higher adapalene retention in the epidermis and dermis compared to the adapalene suspension. NS-HPMC reduced, while NS-HA increased, the accumulation of adapalene in all skin layers. Confocal laser scanning microscopy (CLSM) depicted follicular localization of the fluorescent NSs, with HPMC gel restricting their presence to the stratum corneum and epidermis, while HA gel enhanced penetration to all skin layers. In vitro skin irritation assays using human dermal fibroblasts and in vivo animal tolerability studies showed that the HA gel with adapalene-loaded NSs was a nonirritant formulation. Thus, the findings are promising as they confirmed the effectiveness of NSs in targeting adapalene to hair follicles, and their skin tolerability.

Polymeric nanocarriers, utilizing tyrosine-derived nanospheres (TyroSpheres), were developed for adapalene delivery [113]. TyroSpheres were prepared from PEG5K-b-oligo(DTO-SA)-b-PEG5K, which can self-assemble to form micellar-like spherical-shaped nanocarriers. These nanospheres increased the aqueous solubility of adapalene and reduced its crystallinity within the formulation. In addition, TyroSpheres showed, ex vivo in human cadaver and porcine ear skin, an enhanced adapalene accumulation in the upper skin layers and pilosebaceous unit, where acne originates compared to the marketed Differin^®^ gel. Moreover, in vitro skin irritation studies indicated that the encapsulation of adapalene in TyroSpheres significantly mitigated drug irritancy to monolayer HaCaTs and reconstituted human epidermis (EpiDerm™, MatTek Corp., Ashland, MA, USA) compared to Differin^®^ gel. These findings underscore TyroSpheres as a promising carrier system for delivering hydrophobic drugs to hair follicles and the upper epidermis, while minimizing skin irritation associated with the encapsulated drug.

Adapalene was incorporated into spherical micelles composed of d-α-tocopheryl polyethylene glycol succinate diblock copolymer, which significantly increased its aqueous solubility by approximately 50,000-fold (from <4 ng/mL to 0.2 mg/mL). The skin delivery of adapalene from micelle solutions and gel formulations was comparable and 2- and 10-fold higher compared to Differin^®^ gel and Differin^®^ cream (containing 0.1% (*w*/*w*) adapalene), as shown in vitro in full-thickness porcine and human skin [114]. Micelle solutions and gel formulations facilitated the preferential adapalene delivery to the pilosebaceous unit, with delivery rates of 4.5- and 3.3-fold higher, respectively, than the follicle-free skin biopsies. CLSM confirmed the uniform distribution of adapalene in the hair follicles. Overall, the findings demonstrate that polymeric micelles enable a selective, targeted drug delivery to the hair follicle under finite dose conditions, potentially enhancing therapy of follicular diseases and reducing side effects.

As to polymeric nanocarriers, adapalene was loaded also into PAMAM dendrimers, being further formulated into a gel for potential topical treatment of acne [115]. The follicular and skin targeting efficiency of PAMAM dendrimers and their gel formulation was compared to the commercial gel product, Differin^®^ gel. CLSM revealed that PAMAM dendrimers improved the follicular localization and skin deposition of adapalene. The PAMAM dendrimers’ gel formulation, including lower adapalene doses compared to the commercial product, demonstrated efficient drug accumulation in hair follicles. In vitro cell viability studies indicated the relative safety of G2-PAMAM dendrimers, suggesting potential skin tolerance. Overall, PAMAM dendrimers show promise in targeting drugs effectively, reducing the dose and minimizing side effects in acne treatment.

### 4.4. Tarazotene

Tarazotene is also a retinoid used in the treatment of acne, psoriasis, and sun-damaged skin in the form of topical gels, creams, and foam formulations (e.g., Tazorac^®^, Zorac^®^, and Avage^®^). In marketed preparations, it is used at concentrations of 0.05 and 0.1%. Its topical formulation has many side effects including itching, burning, dryness, redness, stinging, rash blistering, skin discolouration, peeling at the site of application, and low bioavailability. In contrast to tretinoin and adapalene, it is soluble in water, which enables its formulation into topical preparations. Besides the conventional marketed preparations, nanocarriers have also been investigated as tarazotene delivery systems. However, at this stage, there are no available data on the use of trazotene encapsulated in phospholipid vesicles (liposomes), but only in non-ionic-surfactant vesicles (niosomes). Thus, we will represent here the results obtained with tarazotene-loaded niosomes, to make readers familiar with the possibility of using tazarotene-loaded vesicles [85].

#### Tarazotene-Loaded Niosomes

Tarazotene-loaded niosomal gel and tarazotene-loaded nanosponge gel were obtained by the incorporation of niosomes and nansponges into carbomer 940 gel (Table 1). These gels were developed in order to enhance further the solubility and topical bioavailability of tarazotene, and to reduce its side effects. These novel formulations were compared to niosomes, nanosponge, plain gel, and the marketed Tazorac^®^ formulation. The authors reported lower tazarotene in vitro permeation through rat skin and 2–3-times-higher skin retention, and, thus, a higher local accumulation efficiency from niosomal and nanosponge gels compared to the conventional Tazorac^®^ formulation, plain gel, niosomes, and nanosponge. Thus, the authors confirmed the enhanced skin retention of tarazotene when it was applied in nanocarriers, which is of crucial importance in the treatment of skin diseases (such as acne, psoriasis, etc.). The authors did, however, not investigate the irritation potential of the tarazotene-loaded niosomal and nanosponge gels [85]. However, in another study which investigated tarazotene-loaded proniosomes (composed of different ratios of Span^®^ and Tween^®^, cholesterol, lecithin, ethanol, and phosphate-buffered saline) for a possible use in the treatment of psoriasis, skin irritation was also investigated. High drug retention in vitro in rat skin (40–60% of the applied amount) was reported, while no irritation was observed after their in vivo application onto rabbits over 24 h (Draize test) [116]. This finding was very encouraging as one of the aims of tarazotene encapsulation into vesicles is to decrease skin irritation.

## 5. Topical Antibiotics in Treatment of Acne

Antibiotics have been used for the treatment of acne due to the recommendations of various aforementioned guidelines, such as the recommendation of NICE to use fixed topical combinations of clindamicyne with tretinoin or BPO. The updated ADD Guideline also strongly recommends, for the treatment of mild and moderate to severe acne, the use of topical antibiotics based on moderate certainty evidence from 14 studies, as well as the use of fixed-dose topical combinations of BPO and topical retinoid, BPO and topical antibiotic, and topical antibiotic and topical retinoid, based on moderate certainty evidence from seven, nine, and three studies, respectively [3]. However, antibiotic resistance in acne is becoming a significant concern in dermatology since the 1980s. A prominent example is the persistent global issue of resistance to topical erythromycine and clindamycine. In contrast, resistance to systemic treatment with tetracyclines has remained low over the past decade. However, there is a growing trend of resistance to newer systemic macrolides such as azithromycine and clarithromycine [117]. Thus, topical antibiotics are never used as monotherapy in the treatment of acne, but combined with other anti-acne drugs, such as topical retinoids and BPO, as mentioned above. The combined use of topical antibiotic with BPO enhances efficacy and prevents the development of antibiotic resistance [3]. As to oral antibiotics (such as tetracyclines), they are also combined with topical treatments, for moderate-to-severe acne, which do not respond to topical treatment [118]. NICE recommends for moderate to severe acne a combination of oral lymecycline or oral doxycycline with a fixed combination of topical adapalene and BPO, or with topical azelaic acid [6]. The ADD Guideline recommends limiting the use of systemic antibiotics when it is possible in order to reduce antibiotic resistance and other antibiotic-associated complications [3]. When it is necessary, oral tetracyclines, including doxycycline (strong recommendation), minocycline, and saracycline (conditional recommendations), are commonly used. NICE cautions against using oral antibiotics alone for acne treatment. It is recommended to concurrently use BPO and other topical therapies in order to mitigate the risk of antibiotic resistance and to minimize the duration of systemic antibiotic exposure. The systemic antibiotic therapy should be limited to the shortest duration feasible, typically not exceeding 3-4 months, in accordance with international guidelines [3].

### 5.1. Clindamycine

#### Clindamycine-Loaded Conventional and Elastic Liposomes

Clindamycine was one of the first antibiotics encapsulated into liposomes with the aim to enhance its therapeutic effectiveness, while decreasing its side effects. Already, in the year 2000, MLV conventional liposomes encapsulating 1% clindamycine were developed (Table 2). They showed the appropriate stability, enhanced intradermal drug penetration, and enhanced therapeutic effectiveness in the treatment of acne vulgaris in a double-blind clinical study compared to the conventional 1% clindamycin solution (Klimicin T^®^, Lek, Ljubljana, Slovenia). Furthermore, no side effects were reported [119].

Five different clindamycine phosphate liposome formulations, including cationic and anionic liposomes, were prepared (Table 2). The incorporation of the cationic lipid provided a higher stability of the liposome formulation and higher skin permeation of the drug compared to the anionic and neutral liposomes, and especially compared to the control solution. Thus, cationic liposomes could be a promising topical delivery system for clindamycine phosphate for the treatment of acne vulgaris [120].

Clindamycin phosphate was encapsulated into transfersomes, which were incorporated into a carbomer gel. Compared to the control carbomer gel, the transfersomal gel exhibited the significantly higher in vitro release of clindamycine phosphate. Additionally, the transfersomal gel demonstrated a significantly higher cumulative drug permeation and flux in ex vivo rat skin compared to both the transfersome dispersion and the control carbomer gel. This resulted in the enhanced penetration and deposition of clindamycine in the skin, indicating that transfersomes may function as a drug reservoir, extending the effect of clindamycine between successive administrations. The increased skin permeability observed with the transfersomal gel is linked to improved vesicle partitioning into the SC, being a pivotal factor facilitating the release of vesicle-bound drug into the skin. The authors suggest that the clindamycine phosphate-loaded transfersomal gel holds potential as an efficacious carrier for improved dermal delivery of clindamycine phosphate, especially in the treatment of skin infections [121].

### 5.2. Tetracyclines

Among tetracyclines, tetracycline hydrochloride is most often used. It is a broad-spectrum antibiotic and belongs to the tetracyclines-class antibiotics, which stand out as the most extensively researched and commonly prescribed oral antibiotics for acne. Tetracyclines are the only class of antibiotics with US FDA indications specifically for acne treatment. These antibiotics effectively target two pivotal factors in the pathogenesis of acne, demonstrating their antimicrobial effects on *Cutibacterium acnes* and exerting anti-inflammatory mechanisms. The antimicrobial activity is realized through the inhibition of protein synthesis within the bacterial cell [126]. The Guidelines from the American Academy of Dermatology recommend oral antibiotics as a primary treatment for moderate to severe acne, not as monotherapy, but in combination with a topical retinoid and/or a benzoyl peroxide product. This combination approach aims to enhance the effectiveness of acne treatment and reduce the risk of antibiotic resistance [3,126]. These recommendations are given also by NICE, i.e., either oral lymecycline or oral doxycycline should be combined with topical adapalene and BPO, or these oral antibiotics should be combined with topical azelaic acid, for moderate to severe acne [5].

Since tetracycline hydrochloride must be taken on an empty stomach, causing gastrointestinal upset and phototoxicity, its oral application is unfavorable, and, thus, there is a need for advantageous innovative topical formulations loaded with tetracycline hydrochloride.

#### Tetracycline-Hydrochloride-Loaded Conventional and Elastic Liposomes

Hasanpouri et al. have investigated the possibility of using liposomes and transfersomes for the dermal delivery of tetracycline hydrochloride for the treatment of acne [122]. Antimicrobial analysis, which investigated the minimal inhibitory concentration (MIC) values of tested formulations against *Staphylococcus epidermidis,* indicated that there was no difference between vesicles and the aqueous solution of tetracycline hydrochloride. In vitro drug release and ex vivo drug permeation through excised rat skin were performed to assess drug delivery efficiency. The permeation studies showed that the deposited drug amount in the SC was increased by the encapsulation of the drug into transfersomes and liposomes (Table 2), compared to the tetracycline hydrochloride aqueous solution. Despite the higher skin deposition of transfersomal tetracycline hydrochloride compared to the liposomal drug, lower amounts of drug were found in the receptor phase when transfersomes were used compared to liposomes, indicating lower systemic drug absorption, and, thus, less side effects when using transfersomes. Based on these findings, the authors proposed that liposomes and, especially, transfersomes could be a promising delivery system for tetracycline hydrochloride, which would provide better therapeutic effectiveness in treating acne than conventional preparations of tetracycline hydrochloride, while exhibiting less systemic side effects [122].

Liposomes containing tetracycline hydrochloride and tretinoin were developed by Eroğlu et al. In order to obtain a semisolid preparation suitable for topical application, the authors dispersed optimized liposomal formulations in a carbomer gel [123]. An in vitro release study revealed a sustained release of both drugs for 24 hours (Table 2). Cytotoxicity tests confirmed that the liposomal gels were non-toxic. In addition, the antibacterial efficacy of liposomal gels has been confirmed in *Staphylococcus aureus* and *Streptococcus epidermidis* strains, as well as their effect on the biofilm formation and eradication. Thus, it was possible to prepare stable, safe, and efficient liposomal gels containing two active ingredients, one having a comedolytic and the other having bacteriostatic effect, which would enable a therapeutically effective anti-acne treatment using one formulation, thereby leading to improved patient compliance [123].

### 5.3. Roxithromycine

Newer antibiotics have also been investigated for the treatment of acne vulgaris. Roxithromycine, a semi-synthetic macrolide antibiotic, exhibits both bacteriostatic and bactericidal effects on bacterial organisms by interrupting their protein synthesis and growth [127]. Roxithromycine demonstrates a broad spectrum of activity, including effectiveness against *C. acnes*, being responsible for inflammatory acne [128]. A notable challenge associated with roxithromycine is its limited aqueous solubility (0.0335 mg/mL at 25 °C) and unfavorable partition coefficient (*log P* of 2.9), which hinders its therapeutic capabilities. These solubility challenges could be addressed by utilizing various solid-state amorphous forms of roxithromycine, since various crystal lattices of the same compound lead to modified physico-chemical properties, including solubility [129]. Furthermore, solubility issues could be resolved by using different nanocarriers acting also as drug solubilizers, besides acting as percutaneous penetration enhancers. Since there are no studies investigating phospholipid vesicles (liposomes) as carriers for roxithromycine, we represent here other vesicles loaded with roxithromycin.

#### Roxithromycine-Loaded Different Vesicles

Different roxithromycine solid-state forms (i.e., one crystalline and two amorphous forms) were encapsulated into different vesicle systems (niosomes, proniosomes, ufosomes, and pro-ufosomes). Ufosomes, a distinct category of liposomes, are created from fatty acids. These vesicles, composed of fatty acids and ionized (soap) species, form lipid bilayers with an average particle size of 100 nm [124]. Concerning pro-vesicles, they are designed to address stability issues observed in vesicles suspended in aqueous solutions, like niosomes and ufosomes. These systems consist of a dry, porous powder that is water-soluble. Upon the addition of an aqueous solvent before use, pro-vesicles (pro-niosomes and pro-ufosomes) transform into vesicles, specifically niosomes and ufosomes in this case. All vesicles facilitated topical drug delivery to the epidermis and dermis, with no detection of the drug in the receptor compartment and the stratum corneum–epidermis region of the skin. Among these vesicles, niosomes proved to be the most effective formulation for delivering roxithromycin to the dermis, where activity against *Cutibacterium acnes* is required. In terms of different solid-state drug forms, two amorphous forms achieved a higher topical delivery than the crystalline form [124]. Thus, roxithromycin-loaded niosomes could be beneficial for the treatment of acne vulgaris.

### 5.4. Dapsone

Dapsone has also been used to treat acne as it is a sulfone with antibiotic and anti-inflammatory activity. It is commonly used to treat *Mycobacterium leprae*. However, it is also used to treat mild to moderate acne vulgaris. Its use in topical preparations is challenged by its low water solubility and poor skin permeability. Thus, different approaches, such as the use of nanocarriers, are needed to enable its skin delivery, i.e., deposition of therapeutically effective drug amounts in the dermis.

#### 5.4.1. Dapsone-Loaded Invasomes, Niosomes, and Bilosomes

Dapsone was loaded into invasomes, representing phospholipid vesicles containing, besides PC, also terpenes and a small amount of ethanol. Dapsone-loaded invasomes enhanced in vivo in Wistar rats, the skin delivery of dapsone, showing an about-2.5-fold-higher deposited drug amount in the skin compared to the drug solution (Table 2) [125].

Dapsone was encapsulated into different niosomes composed of Span^®^ 20, cholesterol, and PEG-40 hydrogenated castor oil (Cremophor^®^ RH) [130]. The optimized formulation showed in vivo penetration up to the dermis layer. Regarding the therapeutic efficacy, which was investigated in an acne mouse model, the group treated with dapsone-loaded niosomal gel showed significantly better skin recovery and a higher reduction in inflammation percentage compared to the untreated group and the group treated with the ethanolic erythromycine solution (Aknemycin^®^). These results were confirmed by the histopathological examination. Additionally, the bacterial loads were significantly lower in the groups treated with the dapsone-loaded niosomal gel and erythromycine solution compared to the untreated group.

Dapsone was also encapsulated into novel vesicular carriers, i.e., bilosomes, composed of of bile salt (the concentration was varied), and Span^®^ 60:cholesterol used at different molar ratios [131]. The optimal bilosomes contained Span^®^ 60:cholesterol (5:1) and 0.25 M sodium deoxycholate as the bile salt. After 24 h, the skin treated with dapsone-loaded bilosomes retained 170.57 ± 55.12 µg/mL of dapsone, whereas the dapsone-alcoholic solution retained 120.24 ± 10.7 µg/mL, indicating a 1.5-fold-higher drug retention with bilosomes. A histopathological examination of the skin treated with dapsone-loaded bilosomes showed normal structures without inflammation, suggesting their safety and tolerability. These findings highlight the potential of dapsone-loaded bilosomes for improving topical acne therapy.

#### 5.4.2. Dapsone-Loaded Solid Lipid Nanoparticles and Mixed Polymeric Micelles

Deskhar et al. [132] developed SLNs loaded with dapsone. The optimized SLNs were further incorporated into a 1% carbomer (Carbopol^®^ 934) gel, which was investigated for in vitro drug release and ex vivo permeation through rat skin. The dapsone-loaded SLN gel exhibited a biphasic drug release pattern with a significantly higher drug permeation through rat skin, showing a steady state flux (Jss) of 27 ± 2.1 µg/cm^2^/h in comparison to a conventional dapsone gel with a Jss of 22.64 ± 1.8 µg/cm^2^/h.

DAP-loaded polymeric micelles were prepared using Pluronics F-68 and F-127 and the optimized formulation was incorporated into a gel base containing hypromellose (HPMC K100M), sodium carboxymethyl cellulose (NaCMC), and carbomer (Carbopol 980) as gelling agents [133]. Compared to the solubility of free dapsone (0.24 ± 0.056 µg/mL), dapsone solubility in mixed micelles reached 18.42 ± 3.4 µg/mL in water at room temperature. The spreadability order of the gels was found to be NaCMC < HPMC < Carbopol 980. As to the subacute dermal toxicity, studies conducted in rat skin for 21 days revealed no signs of erythema or edema. These findings indicate that mixed micelles can significantly enhance the solubility, permeability, and sustained release of dapsone, while diminishing irritation, making them promising carriers for the dermal delivery of dapsone in anti-acne therapies.

## 6. Miscellaneous Anti-Acne Drugs

Besides retinoids and antibiotics, different pharmaceutical and cosmetic active ingredients, possessing antibacterial and inflammatory activity, are used in the treatment of acne vulgaris.

### 6.1. Benzoyl Peroxide (BPO)

Benzoyl peroxide (BPO) is an effective topical agent for the treatment of acne, which acts by the inhibition of *Cutibacterium acnes* within the pilosebaceous units and on the skin. It exhibits a keratolytic effect in addition to its antimicrobial activity [134], as well as mild anti-inflammatory and comedolytic effects [135]. BPO also exerts mild sebostatic effects. In contrast to topical antibiotics, drug resistance does not develop when BPO preparations are used. It shows the highest effectiveness when it is used combined with other anti-acne-vulgaris drugs. The combination of BPO with either retinoids and topical antibiotics, such as erythromycin or clindamycin, acts synergistically and is well-tolerated. In more severe acne, when oral antibiotics are required, the topical application of BPO can contribute to suppressing the emergence of resistant strains of *C. acnes* [135]. NICE recommends the use of BPO in a fixed combination with adapalene for any acne severity, with clindamycine for mild to moderate acne and with topical adapalene together with oral lymecycline or oral doxycycline for moderate to severe acne [5]. The updated Guideline of the AAD from the year 2024 strongly recommends BPO for mild and moderate to severe acne treatment based on moderate certainty evidence [3]. However, this guideline recommends, like other guidelines, a multimodal therapy with topical antibiotic or topical retinoid as a good practice statement to optimize the efficacy of the treatment, as well as to reduce the risk of antibiotic resistance [3]. Besides being the preferred first-line therapy for the treatment of mild to moderate acne, it shows side effects, such as peeling, itching, redness, dryness, burning, and dermatitis, which may limit patient compliance [7,136]. However, in combination with a topical antibiotics, BPO produces less erythema than when used alone. Marketed products such as gels and washes contain BPO in the concentration range from 2.5 to 10% (*w*/*v*). As to physico-chemical properties, it is poorly soluble in water and many commercially available formulations often contain insoluble BPO crystals, which trap BPO in the interior of the clusters, thus reducing bioavailability. Therefore, solubilized BPO lotions have been developed [137]. In addition, BPO is also prone to degradation and is unstable in solution [138]. The stability and solubility limitations, together with the adverse effects of BPO, make it a suitable candidate for liposomal entrapment, which overcomes all these limitations.

#### 6.1.1. Benzoyl-Peroxide-Loaded Conventional Liposomes

One of the first studies on the encapsulation of BPO into liposomes was performed already in 1999. This in vivo study, which used infundibular bacterial samples from 20 acne patients, revealed that BPO encapsulation into liposomes indeed significantly increased its antibacterial effect in the infundibula against *Propionibacteria* and *Micrococcaceae* compared to a commercial product and pharmacopoeial formulation of BPO (Table 3). Thus, treatments with liposomal BPO represent an improvement of the conventional topical treatments of acne with BPO [139].

In a next study, BPO was loaded into liposomes, which were further incorporated into a carbomer gel. The obtained liposomal gel was compared to a plain non-liposomal BPO gel in an in vivo comparative double-blind study in 30 patients [140]. Liposomal gel demonstrated a significant improvement in the therapeutic response of acne after 3 months of treatment as compared to the conventional BPO gel, as well as a marked reduction in local irritation. The authors compared these effects to the effects of liposomal tretinoin gel from their earlier studies, and concluded that tretinoin and BPO liposomal gels led to a similar reduction in the total number of skin lesions (Table 3).

**Table 3:** Vesicles used as carriers for miscellaneous anti-acne drugs.

Formulation	Drug	Therapeutic Indication	Experimental Condition	Outcome	Reference
**Benzoyl-peroxide-loaded liposomes in clinical studies**					
BPO-liposomes compared to BPO-commercial product and pharmacopoeial formulation of BPO	Benzoyl peroxide (BPO)	Acne vulgaris	In vivo, in humans, single-blinded, comparative study, two-week, twice-daily treatment with test formulations, 20 patients	Infundibular bacterial samples were collected using the Permabond technique from 20 acne patients subjected to a two-week, twice-daily treatment regimen on the upper back with the test formulations. The treatments were vehicle-controlled. Liposomes significantly increased the antibacterial effect of BPO in the infundibula against *Propionibacteria* and *Micrococcaceae* compared to other formulations.	[139]
Liposomes were incorporated into a carbomer (carbopol 934) gel; Plain non-liposomal BPO gel	Benzoyl peroxide	Acne vulgaris	In vivo, in humans, double-blinded, comparative study, 30 patients, 3 months	After 3 months of treatment, the liposomal gel exhibited a significant enhancement in the therapeutic response for acne, approximately being 2-fold the effectiveness of the conventional BPO gel. Liposomal BPO-gel significantly decreased drug penetration. BPO-liposomal gel led to a notable decrease in local irritation.	[140]
Simultaneous application of BPO (2.5%)- and tretinoin (0.025%)-liposomal carbomer gels on one face side, and conventional carbomer gels of the same drug concentration on the other side (BPO in the morning and tretinoin in the evening).	Benzoyl peroxide Tretinoin	Acne vulgaris	In vivo, in humans, double-blinded, comparative study, 30 patients, 3 months	Dual therapy with liposomal gels provided an almost complete cure (100% reduction in skin lesions: comedones, papules, and pustules) within 10 weeks, while, with the conventional gels containing BPO and tretinoin, 75% to 80% reduction in skin lesions was achieved even after prolonging the therapy for up to 12 weeks. Liposomal gels demonstrated superior outcomes across various skin lesions within 8 weeks, a result not achieved even after 12 weeks of conventional gel treatment. Combined therapy with liposomal gels of tretinoin and benzoyl peroxide also reduced adverse effects of the drugs, which increased patient compliance.	[141]
**Benzoyl-peroxide- and chloramphenicol-loaded liposomes**					
Different liposomes (soybean lecithin, propylene glycol, and distilled water (2:1:2 *w*/*v*/*v* ratio)) were prepared: BPO-CAM-liposomes BPO-liposomes CAM-liposomes, and empty liposomes	BPO and chloramphenicol (CAM); co-encapsulation	Acne vulgaris	In vitro, drug release studies	CAM exhibited a sustained release from all liposomes, while BPO appeared to be retained within the liposomes, possibly due to its poor water solubility.	[142]
			In vitro, MTT assay on HaCaT cells, final sample concentrations: 0.1–4.0% (*v*/*v*), corresponding to between 5 to 200 µg/mL CAM and 0.75 to 30 µg/mL BPO	BPO in all formulations gave the most prominent effect of the cell viability. Liposomal BPO showed lower toxicity in comparison to BPO solution. Liposomal CAM showed a higher toxicity than CAM solution. Presence of CAM in liposomes did not affect the cytotoxicity of BPO; i.e., BPO liposomes and BPO solutions exhibited similar toxicity like CAM-BPO liposomes and CAM-BPO solution. At lower concentrations (<4%), CAM-BPO liposomes were more cytotoxic than those with only BPO, especially compared to drug solutions. However, at higher concentrations (4%), both BPO and BPO-CAM liposomal formulations showed similar, reduced cytotoxicity compared to control drug solutions.	
**Benzoyl-peroxide- and tretinoin-loaded niosomes**					
Tretinoin-niosomes, and BPO-niosomes incorporated into a carbomer gel (0.020% tretinoin and 0.600% BPO) W/O anti-acne cream and alcoholic solution (containing the same drug concentrations)-control	BPO Tretinoin	Acne vulgaris	In vitro, Wistar rat skin	Niosomal gel provided after 24 h, lower cumulative permeated amounts of tretinoin and BPO compared to controls.	[143]
			In vivo, Wistar rats	The use of the niosomal gel, anti-acne cream, and alcoholic solution delivered amounts of 15.54 μg, 11.54 μg, and 2.68 μg of tretinoin, and amounts of 143.78 μg, 68.85 μg, and 59.98 μg of BPO to the skin, respectively.	
			In vivo, in rabbit ear pinna model	Niosomal gel with a 4.16-fold-lower dose of BPO provided the same therapeutic index at targeted sites as the anti-acne cream. Histopathology micrographs showed that no comedones were present in the treated pinnas after a 14-day treatment with both anti-acne cream and the niosomal gel.	
**Azelaic-acid-loaded liposomes**					
PC-liposomes and ethosomes with varying concentrations of ethanol (20–45% *v*/*v*), incorporated into carbomer gels	Azelaic acid (AA)	Acne vulgaris	In vitro, cellulose ester-based membrane	The highest diffusion coefficients were found for ethosomes containing 40% *v*/*v* of ethanol. The diffusion coefficients from gels were lower compared to dispersions.	[144]
Azelaic acid (AA), AA complexed with hydroxypropyl-beta-cyclodextrin (AACD), AA esterified to diethyl azelate (DA) Three forms of AA were encapsulated into liposomes (L-alpha-dipalmitoyl phosphatidylcholine/cholesterol = 7:3) and niosomes (Tween 61/cholesterol = 1:1).	Azelaic acid in three forms	Acne vulgaris	In vitro, cytotoxicity test, in mouse epidermal cell lines (JB6, normal cell lines) by the sulforhodamine B assay	Liposomes and niosomes, containing AA, showed modest cytotoxicity when compared to cisplatin. Empty liposomes and niosomes gave no growth inhibitory effect.	[145]
			In vivo, in rabbit skin	No signs of erythema or edema in rabbit skin within 72 h, when the three forms of AA were encapsulated.	
Liposomal hydrogel, and commercially available cream (20% AA)	Azelaic acid	Acne vulgaris	In vitro, pig ear skin	Liposomal gel provided very high accumulation of AA in the SC (187.5 μg/cm^2^), which enables a decrease of the AA concentration in the liposomal gel to 10% in comparison to the marketed cream containing 20% of azelaic acid.	[146]
			In vitro	High antibacterial activity of the liposomal gel was shown, eliminating the need for additional preservatives.	
Invasomes, liposomes, and LeciPlex: cetyltrimethylammonium bromide (CTAB) Leciplex and didodecyldimethylammonium bromide (DDAB) Leciplex	Azelaic acid	Acne vulgaris	In vitro, skin penetration	Invasomes showed highest penetration of azelaic acid.	[147]
			In vivo, in rats	Invasomes with AA produced the highest anti-acne efficacy, followed by liposomes and LeciPlex.	
			In vitro, *C. acnes*	AA showed high antimicrobial activity when it was encapsulated in DDAB LeciPlex, followed by almost equal activity for invasomes and CTAB LeciPlex, followed by liposomes.	
**Trehalose-loaded liposomes in clinical study**					
Ready-to-use peel-off facial mask containing trehalose-loaded liposomes and myoinositol	Trehalose Myoinositol	Acne vulgaris	In vivo, in humans, application for 60 days, every second day, overnight	The mean counts of comedones, papules, pustules, and nodular lesions decreased significantly, as well as the sebum production and the mean Global Acne Grading System (GAGS) scale score. Decreased levels of testosterone and dehydroepiandrosterone sulfate in skin biopsy supernatants were found, while levels of beclin-1 (a marker of authophagy) increased significantly.	[148]
**Lauric-acid-loaded liposomes**					
Lauric acid, palmitic acid, and oleic acid encapsulated in egg PC:cholesterol liposomes	Lauric acid (LAH) Palmitic acid Oleic acid	Acne vulgaris	In vitro, *C. acnes*	Of the three free fatty acids, LAH exhibited the most potent bactericidal activity against *C. acnes*. LAH exhibited higher antimicrobial activity when encapsulated in liposomes even at low LA concentration. The LAH activity in liposomes were mainly dependent on the LAH concentration in one single liposome.	[149]
Dipalmitoylphosphatidylcholine (DPPC)-liposomes with LAH (50 mol%)	Lauric acid (LAH)	Acne vulgaris	In vitro (ex vivo), pig ear skin	LAH, when integrated into the vesicles, exhibited minimal penetration through the skin at both pH levels (pH 7.4 and 5.0), indicating that the primary sites of LAH deposition occurred within the layers of the skin.	[150]
Lauric acid-liposomes, curcumin-loaded liposomes, and azithromycin-loaded liposomes were incorparated into carbomer gels	LAH Curcumin Azithromycin	Acne vulgaris	In vitro, agar diffusion assay	The lauric-acid-liposomal gel demonstrated an approximately 1.5-fold increase in antibacterial effectiveness compared to the curcumin-liposomal gel. Co-application of these liposomal gels in equal proportions (1:1) resulted in a significantly amplified antibacterial effect against both macrolide-sensitive (1.81- versus 1.25-fold) and -resistant strains of C. acnes (2.93 versus 1.22-fold) in comparison to using the individual liposomal gels.	[151]
			In vivo, in rat ear model	The co-application of the two liposomal gels resulted in an approximately 2-fold reduction in the number of comedones and levels of cytokines (TNF-α and IL-1β) compared to the placebo-treated group.	
**Cryptotanshinone (CPT)-loaded ethosomes**					
Ethosomes incorporated into a carbomer (Carbopol 974) and conventional hydroethanolic gel	Cryptotanshinone (CPT)	Acne vulgaris	In vitro, pig skin	Ethosomal gels demonstrated approximately 2.5 times the cumulative permeation and transdermal flux compared to the conventional gel. The skin deposition of CPT using ethosomal gels was measured at 1.330 ± 0.344 μg/g, representing a 2.1-fold increase compared to the control, indicating that ethosomes enhance the deposition of CPT into the skin.	[152]
			In vivo, oleic-acid-induced acne model in rabbits	Ethosomal gels loaded with CPT exhibited a more effective anti-acne outcome compared to conventional gels, and rabbits treated with CPT-loaded ethosomal gel showed a near-complete restoration of normal skin structure	
			In vivo, irritation test	No visual dermal reactions, such as erythema or edema, were observed in either the CPT ethosomal gel or the blank gel treated groups. The primary irritation indices at each time point remained 0, confirming no skin irritation for the CPT ethosomal gel. Histological examination revealed similar skin structures between the CPT ethosomal gel and blank ethosomal gel groups, resembling untreated skin.	
**Rosmarinic-acid-loaded niosomes**					
Niosomes (Span^®^ 85 and cholesterol) were incorporated into a carbomer gel → niosomal gel Plain drug gel Marketed BPO gel	Rosmarinic acid (RA)	Acne vulgaris	In vitro, *P. acne* and *S. aureus*	Niosomes and niosomal gel demonstrated higher antibacterial activity in comparison to plain gel.	[153]
			In vivo, Swiss albino mice, against *P. acne* only	Niosomal gel showed activity against *P. acnes* until the fourth day of application due to the prolonged drug release from niosomes, in contrast to the marketed gel and plain solution of rosmarinic acid which were effective only on the first day, while no response was observed on the fourth day.	
			In vivo, Swiss albino mice, skin irritation test	Niosomal gel formulation was significantly less of an irritant than the plain rosmarinic acid solution (values between 0 and 9 indicate that there is no irritation). The mean irritation score was as follows: Niosomal gel: 2.13 ± 0.31, and plain RA solution: 4.57 ± 0.62.	
			In vivo, Swiss albino mice, antiinflammatory studies	RA-loaded niosomal gel induced higher inhibition of inflammation than the plain drug solution: Niosomal gel: up to 58.16% in 3 days, plain RA solution: up to 40.9% in 3 days.	
			In vitro drug release	Niosomal gel released after 24 h: 49.81 ± 1.76% of the drug.	
			In vitro, Swiss albino mice skin, skin permeation	Niosomal gel: 48% of drug permeated and 35% of drug retained in the skin, plain gel of RA: 21% of drug permeated and 15% of drug retained in the skin.	
**N,N-diethyl-4-methyl-3-oxo-4-aza-5α-androstane-17β-carboxamide (4-MA)-loaded liposomes**					
4-MA-liposomes and 4-MA-solution	N,N-diethyl-4-methyl-3-oxo-4-aza-5α-androstane-17β-carboxamide (4-MA)	Acne vulgaris	In vivo, hamster	4-MA induced apoptosis and inhibited the growth of dihydrotestosterone (DHT)-dependent hamster flank organ sebaceous glands. Only 4-MA-liposomes showed selective efficacy. Further, liposomes encapsulating 4-MA did not significantly impact prostate weight, testosterone/DHT ratios, or bodyweight gain compared to controls.	[154]
**Cyproterone-acetate-loaded liposomes**					
Liposomes of different lipid content and different particle size, Derma membrane structure (DMS) creams, and phospholipid concentrate	Cyproterone acetate (CPA)	Acne vulgaris	In vitro, dermatomed porcine skin	Increase in lipid content increased the CPA skin permeation. In addition, the decrease of liposomal particle size also increased the skin permeation. As to other formulations, cumulative drug amount permeated (up to 48 h) from the DMS creams was 2.9–6.8 μg/cm^2^. When the phospholipid concentrate was used, the CPA permeation was 2.6-fold-increased in comparison to the control DMS cream.	[155]
Liposomes (of different PC:Cholesterol ratio) were incorporated into a hydroxyl ethylcellulose gel → liposomal gel Conventional CPA gel	Cyproterone acetate (CPA)	Acne vulgaris	In vitro, permeation study, abdominal guinea pig skin	Liposomal gel provided higher skin permeation of CPA than the conventional CPA gel. Liposomal gel: approx. 50–60 μg/cm^2^ (cumulative drug permeated), after 6 h Plain gel: approx. 30 μg/cm^2^, after 6 h.	[156]
**PDT and photosensitizer-loaded liposomes**					
Pheo A in transfersomes (1,2-distearoyl-sn-glycero-3-phosphoethanolamine-N-[amino(polyethylene glycol)-2000], cholesterol, Tween^®^ 80) labelled as DSPE-PEG-Pheo A (DPP) transfersomes Pheo A solution Pheo-A-conventional liposomes	Pheophorbide A (Pheo A) as photosensitizer (PS)	Acne vulgaris	In vitro, penetration studies in normal mice skin and *P. Acnes*-induced mice skin	Among different formulations, Pheo-A-transfersomes led to fluorescence only in the dermis of P. acnes-induced skin, while Pheo A solution and Pheo-A-loaded liposomes, delivered Pheo A to the SC or to all skin layers	[157]
			In vivo, nude mice with induced *P. acnes*	Transfersomes were therapeutically effective against acne, as shown by a reduction in swelling volume and skin thickness.	
**PDT and photosensitizer-loaded liposomes in clinical studies**					
0.1% MB-liposomal hydrogel	Methylene blue (MB)	Acne vulgaris	In vivo, in humans, mild-to-moderate acne vulgaris in a randomized, controlled and investigator-blinded study, treatments once a week for two weeks	Liposomal MB gel resulted in complete destruction of the pilosebaceous unit, while the gel with free MB caused only superficial destruction. After two treatments, once a week, liposomal gel of MB induced a reduction of the number of inflammatory acne by 83.3%, while reduction of the number of non-inflammatory acne was 63.6%. Liposomal gel induced no pain, and no serious adverse side effects or edema. After 12 weeks, 90% of patients exhibited a moderate-to-marked improvement in the treated areas.	[158]
0.1% MB-loaded liposomal gel in combination with PDT versus intense pulsed light (IPL) alone	Methylene blue (MB)	Acne vulgaris	In vivo, truncal acne vulgaris, 35 patients, treatment once per week, three sessions with MB-liposomal hydrogel applied on the randomly selected one side of the back, and, after 60 min, the entire back was exposed to IPL. Acne were re-evaluated 1 month after the third session by two independent dermatologists.	At the back side treated with the MB-liposomal gel, inflammatory acne lesion counts were significantly decreased by 56.40% compared to 34.06% achieved with IPL alone. Significant overall improvement was seen on the back side pretreated with MB-liposomal gel in 11.5% of patients compared to 2.8% in the case of IPL treatment performed without pretreatment with the MB-liposomal gel.	[159]
Soya PC-liposomes (0.5% 5-ALA liposomal spray, Ellipse Photo Spray, Ellipse A/S, Denmark) combined with intense pulse light (IPL)	5-aminolevulinic acid (5-ALA)	Acne vulgaris	In vivo, humans	The average reduction in total acne lesions was substantial (68.2%), post-treatment fluorescence was minimal (indicating a reduced risk of phototoxicity), and side effects were minimal following the application of liposomes. The observed effectiveness was comparable to that of 20% 5-ALA creams. The utilization of PC-liposomes was hypothesized to enhance the skin penetration of 5-ALA and its accumulation in the pilosebaceous units, leading to an improvement in acne lesions.	[160]
0.5% liposome-encapsulated 5-ALA spray with intense pulsed light reduced inflammatory lesions	5-aminolevulinic acid (5-ALA)	Acne vulgaris	In vivo, 12 Asian patients	0.5% liposome-encapsulated 5-ALA spray with intense pulsed light reduced inflammatory lesions of 52% at 1 month and 65% at 6 months after treatment of inflammatory facial acne.	[161]

However, liposomal tretinoin gel was found to be more effective in treating comedones, while liposomal BPO gel was more effective in treating papules and pustules, and both gels reduced local side effects, leading to increased patient compliance and, finally, to enhanced therapeutic effectiveness [140].

In previous studies, it has been shown that the liposomal tretinoin gel was significantly more effective in treating comedones, while liposomal BPO gel was more effective in treating papules and pustules, as well as that the adverse effects, such as erythema, itching, burning, scaling, and irritation were less common than when patients were treated with conventional formulations [76,140]. Thus, the same group of authors [141] decided to investigate the benefits of the simultaneous application of tretinoin and BPO liposomal gels, since an additive effect could be expected due to the comedolytic activity of tretinoin and bacteriostatic activity of BPO, as well as a decrease in side effects. The authors performed an in vivo study in patients with mild to moderate acne. The obtained results confirmed the promising role of the simultaneous application of liposomal tretinoin and BPO gels in acne patients compared to conventional gels of BPO and tretinoin. Liposomal gels enabled complete symptom remission in a shorter treatment duration due to the enhanced skin retention of encapsulated drugs (Table 3). The simultaneous use of two liposomal gels exhibited a significantly improved (1- to 1.5-fold) therapeutic response for all acne types, reducing treatment duration and enhancing patient compliance compared to single tretinoin or BPO therapy. Furthermore, the combined therapy with liposomal gels of tretinoin and BPO mitigated adverse drug effects, contributing to increased patient compliance [141].

An interesting strategy involves encapsulating multiple drugs within a single nanocarrier formulation, such as liposomes. This approach aims to achieve a synergistic drug effect and simplify dosing schedules in multi-drug treatment regimens, especially in the topical treatment of acne, which typically involves a combination therapy with two or more drugs, often leading to low patient compliance. This approach allows for the simultaneous and simple delivery of multiple drugs to the target site, leading to enhanced patient compliance, and therapeutic effectiveness.

In order to achieve the co-encapsulation of BPO and chloramphenicol (CAM) into liposomes, Ingebrigtsen et al. developed a novel fast and easy liposome-processing method—dual asymmetric centrifugation (DAC) [142]. This approach circumvented the limitation of CAM, i.e., its systemic toxicity, since liposomes enhance dermal, but decrease systemic, drug delivery. In vitro drug release studies revealed that CAM exhibited a sustained release from all liposomal formulations, while BPO appeared to be retained within the liposomes, possibly due to its poor water solubility (Table 3). However, the cell viability assay showed that BPO is released from the nanocarrier as BPO in all formulations gave the most prominent effect of the cell viability, decreasing it. Liposomal formulations incorporating BPO exhibited reduced toxicity compared to the BPO solution. This suggests that liposomes are able to protect HaCaT cells from BPO, likely attributed to a delayed release of the drug from the carrier, which diminishes the interaction between BPO and the cells. In contrast, liposomes did not show a protective effect when only CAM was incorporated into the liposomes; i.e., the liposomal CAM formulation showed a higher toxicity than CAM solution. Thus, the authors proposed that, for drugs that exhibit no cytotoxicity (CAM), the toxicity of the carrier is more relevant, while, for more cytotoxic drugs (BPO), the protective effect of the carrier towards the drug’s action on the cells is more pronounced than the toxicity of the carrier [142]. Furthermore, the presence of CAM in liposomes did not affect the cytotoxicity of BPO, since both BPO liposomes and BPO solutions exhibited a similar toxicity as CAM-BPO liposomes and CAM-BPO solution. The absence of CAM cytotoxicity, shown here, makes it a promising topical antibiotic for the treatment of acne. Furthermore, i.e., at lower concentrations (<4%, e.g., 1% and 2%, *v*/*v*), CAM-BPO liposomes displayed higher cytotoxicity compared to liposomes containing only BPO, particularly when compared to drug solutions. However, at higher concentrations (4%, *v*/*v*), where the overall cell viability decreased for all samples, liposomal formulations (BPO and BPO-CAM) exhibited similar cytotoxicity, albeit lower than that observed with control drug solutions. The authors reported also that the toxicity of both drugs were independent of their presence as single- or dual-drug formulations and, thus, this dual drug liposomal formulation could be promising in treating acne [142].

#### 6.1.2. Benzoyl-Peroxide-Loaded Niosomes

Similarly, as mentioned above, a combination of tretinoin (keratolytic agent) and BPO (potent antibacterial agent) was used, by encapsulating them separately into niosomes, being further incorporated into a carbomer gel [143]. The in vitro permeation study revealed that the obtained niosomal gel with tretinoin and BPO provided, after 24 h, lower cumulative permeated drug amounts than the W/O anti-acne cream and alcoholic solution (Table 3). However, the niosomal gel provided in vivo in Wistar rats significantly higher drug amounts in the skin compared to the conventional formulations. Further, the in vivo study in the rabbit ear pinna model showed that the niosomal gel was more efficacious than the anti-acne cream containing tretinoin and BPO, since the niosomal gel containing a lower BPO dose provided the same therapeutic effectiveness at the targeted sites as the anti-acne cream (Table 3). Thus, tretinoin and BPO encapsulated in niosomes have proven to be superior in terms of therapeutic effectiveness compared to a conventional anti-acne cream containing these drugs at the same concentrations [143].

### 6.2. Azelaic Acid

Azelaic acid is a derivative of oleic acid possessing anti-keratinizing, as well as bacteriostatic, activities against both aerobic and anaerobic bacteria [162]. Due to its keratolitic, anti-inflammatory and antibacterial activity, it is efficient for the treatment of acne vulgaris; i.e., it is effective in the treatment of comedones and inflammatory lesions [139]. Compared to topical retinoids (such as tretinoin and adapalene) and benzoyl peroxide, it shows a trend towards a better tolerability, i.e., its safety profile [163]. To increase its efficacy and decrease its side effects, it has been incorporated into different nanocarriers, such as liposomes, polymeric nanoparticles, nanocrystals, etc. [164,165]. The ADD Guideline conditionally recommends azelaic acid for the treatment of acne based on moderate certainty evidence from three clinical studies [3].

#### Azelaic-Acid-Loaded Conventional and Elastic Liposomes

Azelaic acid was encapsulated into conventional PC-liposomes and different deformable ethosomes, being further incorporated into carbomer gels (Table 3). The drug diffusion from vesicle dispersions and the aforementioned gels was studied in vitro through a cellulose ester-based membrane and characterized by a steady-state flux. Ethosomes containing 40% *v*/*v* of ethanol provided the highest diffusion coefficients, and ethosomes in general showed higher diffusion coefficients than gels, as vesicles are supposed to be tightly packed in the viscous gel structure compared to liquid dispersions [144]. Thus, a lower skin penetration from liposomal and ethosomal gels compared to liposome and ethosome dispersiones is expected since it has been shown that, with increasing the viscosity of liposomes by adding viscosity modifiers, leading to liposomal gels, the skin penetration of the encapsulated drugs decreases [166].

Panyosak et al. investigated the safety of azelaic acid (AA) and its derivatives being encapsulated into different vesicles [145]. The hydrophilic nature of azelaic acid was altered by complexing it with hydroxypropyl-beta-cyclodextrin (AACD), while its lipophilic property was enhanced through the esterification to diethyl azelate (DA). All three drugs were encapsulated in both liposomes and niosomes. Cytotoxicity tests affirmed their low cytotoxic effects compared to cisplatin (Table 3). Additionally, empty liposomes and niosomes exhibited no inhibitory effect on growth. Regarding the irritation potential of azelaic acid, AACD, or DA, the results indicated no signs of erythema or edema in rabbit skin within 72 h, when they were encapsulated. Therefore, this study confirmed that azelaic acid and its derivatives were safe for topical use when encapsulated in vesicles [145].

In an attempt to enhance the topical bioavailability of azelaic acid, Burchacka et al. developed an azelaic-acid-loaded liposomal hydrogel and compared it to a commercially available product [146]. The novel liposomal gel exhibited satisfactory physical characteristics and facilitated a substantial accumulation of azelaic acid in the SC, allowing for a reduction in the azelaic acid concentration within the gel to 10%, as opposed to the marketed cream containing 20% azelaic acid (Table 3). Moreover, in vitro antimicrobial preservation studies demonstrated high antibacterial activity of the liposomal gel, eliminating the need for additional preservatives in the final formulation. The study not only confirmed the stability and potent antibacterial activity of the liposomal gel with encapsulated azelaic acid, but also highlighted its improved topical bioavailability, presenting a promising drug delivery system for the treatment of acne [146].

Three vesicular systems, deformable invasomes, conventional liposomes, and cationic LeciPlex, were loaded with azelaic acid and compared for their therapeutic efficacy (Table 3). Invasomes loaded with azelaic acid exhibited the best anti-acne efficacy in vivo in rats, followed by liposomes and LeciPlex. As to the in vitro antimicrobial study of azelaic acid loaded in different nanocarriers against *Cutibacterium acne*, it revealed high antimicrobial activity when it was encapsulated in DDAB LeciPlex, followed by almost equal activity for invasomes and CTAB LeciPlex, followed by liposomes [147].

### 6.3. Trehalose-Loaded Conventional Liposomes

Increased androgen levels and diminished skin autophagy are suggested factors in the development of adult female acne. Thus, Fabbrocini et al. tested in female adults with *acne* a ready-to-use peel-off facial mask containing trehalose-loaded liposomes (as activators of cutaneous autophagy) and myoinositol (an androgen inhibitor) (Table 3). The mask improved the cosmetic appearance of acne after 60 days of treatment by reducing cutaneous androgen content and promoting skin autophagy [148].

### 6.4. Lauric-Acid-Loaded Conventional Liposomes

Yang et al. showed that lauric acid (LAH) used alone, as well as in liposomes, possesses the strongest bactericidal activity against C. acnes among three fatty acids (lauric acid, palmitic acid, and oleic acid) (Table 3) [149]. Namely, since LAH is insoluble in water, it was incorporated into liposomes, which additionally enhanced its antimicrobial effect even at a low LA concentration. The mechanism of the antimicrobial activity of LAH-liposomes has been explained by their fusion with the membranes of *C. acnes*, leading to the release of LAH in the membranes of bacteria. These phenomena effectively kill *C. acnes*. Since LAH is a natural compound from coconut oil, LAH liposomes represent a safe, non-irritating, but effective formulation for the treatment of acne vulgaris.

Farkuh et al. developed and characterized vesicles of dipalmitoylphosphatidylcholine (DPPC) containing lauric acid (LAH) for the potential treatment of acne, since LAH has been previously shown to possess antimicrobial activity against *Cutibacterium acnes*. Liposomes provided a minimal permeation of lauric acid through the skin, indicating that the lauric acid was mostly deposited in the skin layers (Table 3). This was desired as the drug should act in the skin and have no effect at the systemic level. The authors also demonstrated that lauric-acid-containing lipid vesicles may incorporate water soluble probes, enabling, at the same time, the delivery of lauric acid, being an antimicrobial compound, and a water-soluble active compound for the treatment of acne, leading to synergistic effects in the treatment of acne [150].

Madan et al. investigated different liposomal gels obtained by the incorporation of lauric-acid-loaded liposomes, curcumin-loaded liposomes, and azithromycin-loaded liposomes into carbomer gels [151]. As to their antibacterial activity, the lauric-acid-liposomal gel was more efficient than the curcumin-liposomal gel (Table 3). Furthermore, the combined application of these liposomal gels (1:1) demonstrated a significantly increased antibacterial effect against both macrolide-sensitive and -resistant strains of *C. acnes*, when compared to using the individual liposomal gels. Regarding the therapeutic efficacy, the combined use of both liposomal gels decreased the number of comedones and cytokine levels (TNF-α and IL-1β) compared to the placebo-treated group. These findings underscore the importance of co-applying bioactive components, such as curcumin and lauric acid, loaded in liposomal gel formulations for the effective treatment of acne vulgaris.

### 6.5. Cryptotanshinone-Loaded Ethosomes

Cryptotanshinone is a well-known diterpene quinone from the widely used traditional Chinese herb *Salvia miltiorrhiza*, which possesses various pharmacological effects, including anti-inflammatory, anticancer, and cardio-cerebrovascular protection activities [167]. Cryptotanshinone is of low water solubility, and, thus, formulation approaches are needed to overcome this problem. There are various nanotechnology approaches used to enhance the efficacy and mitigate the adverse effects of herbal medicines, such as the use of polymeric micelles, nanoemulsions, microspheres, polymeric nanoparticles, nanocapsules, liposomes, phytosomes, transferosomes, and dendrimers, as well as ethosomes [168]. In this study, cryptotanshinone was encapsulated into ethosomes, being further formulated into a topical carbomer gel for the treatment of acne, which was compared to a conventional hydroethanolic gel. The in vitro obtained transdermal flux and skin deposition of the active ingredient encapsulated in the optimized ethosomal gel were 2.5- and 2.1-times higher compared to a conventional gel. As to the anti-acne activity and skin irritation in rabbits, the ethosomal gel revealed a better anti-acne effect with only slight skin irritation, indicating its potential to be used in the treatment of acne (Table 3) [152].

### 6.6. Salicylic-Acid-Loaded Liposomes

Salicylic acid (SA) is a beta-hydroxy acid, which is often applied for the treatment of acne. It has been shown to decrease sebocyte lipogenesis, reduce inflammation, and suppresse the levels of cytokines and major pathogenic proteins around acne lesions [169]. It also shows comedolytic activity and is available on the market in formulations with concentrations in the range of 0.5–2% [3]. Thus, it has been used frequently in the treatment of acne combined with other anti-acne drugs [170,171,172]. The ADD Guideline conditionally recommends the use of salicylic acid for the treatment of acne based on moderate certainty evidence from one clinical study [3]. However, it suffers from the disadvantage of being a mild to strong irritant. This drawback can be circumvented through its encapsulation into liposomes. Bhalerao and Harshal succeeded in developing salicylic-acid-loaded liposomes possessing adequate stability when stored at 4–5 °C [173]. However, they did not investigate their therapeutic effectiveness, nor their irritation potential. Therefore, studies are needed to evaluate their efficacy in treating acne, as well as their skin irritation potential.

### 6.7. N,N-Diethyl-4-methyl-3-oxo-4-aza-5α-androstane-17β-carboxamide (4-MA)-Loaded Liposomes

Li et al. developed a liposomal hair-follicle-targeting system containing a selective 5-α-reductase inhibitor to address androgen-dependent acne pathology. The aim was to modify pathological processes in the pilosebaceous unit, as observed in acne vulgaris [154]. The authors demonstrated that the liposomal formulation incorporating the 5-α-reductase inhibitor N,N-diethyl-4-methyl-3-oxo-4-aza-5α-androstane-17β-carboxamide (4-MA) induced apoptosis and inhibited the growth of dihydrotestosterone (DHT)-dependent hamster flank organ sebaceous glands. A comparison between liposomes with encapsulated 4-MA and the 4-MA solution showed selective efficacy only for the liposomal formulation (Table 3). Apoptosis induced by liposomal 4-MA was confirmed through DNA fragment assays and the observation of condensed and fragmented nuclei. Moreover, liposomes encapsulating 4-MA did not significantly impact prostate weight, testosterone/DHT ratios, or bodyweight gain compared to controls, indicating both safety and efficacy in addressing pathological processes like acne vulgaris [154].

### 6.8. Steroid-Hormone-Loaded Liposomes

Steroid hormones, cyproterone acetate (CPA) and finasteride, have also been used in the topical treatment of acne vulgaris. However, these drugs show low skin permeability, which limits their therapeutic effectiveness due to insufficient drug penetration into the skin. It has been shown that these steroid hormones, as well as 17-beta estradiol, can be encapsulated into liposomes (DPPC) [174]. In another study, in vitro permeation through dermatomed porcine skin revealed that cyproterone acetate permeation from liposomes was strongly dependent on the lipid content. The higher the lipid content was, the higher the CPA skin permeation was. The decrease of the liposome particle size by extrusion resulted in a two-fold increase in CPA permeation compared to the unextruded liposomes. This study also confirmed the possibility of controlling the CPA skin permeation by using different formulations (liposomes, phospholipid concentrate, and Derma membrane structure creams), which contain different phospholipids with saturated and unsaturated fatty acids (Table 3) [155].

In another study, CPA has been loaded into liposomes with different PC:Cholesterol ratios. Afterwards, the optimal CPA-liposomes were incorporated into a hydroxyl ethylcellulose gel, as well as the free CPA, in order to obtain a liposomal and a conventional gel, respectively. The results of the in vitro percutaneous permeation study through the abdominal skin of guinea pigs showed that the liposomal gel enabled a higher skin permeation of CPA than the conventional CPA gel (Table 3) [156].

### 6.9. Rosmarinic-Acid-Loaded Niosomes

The resistance of *Staphylococcus aureus* and *Propionibacterium acnes*, i.e., bacteria which colonize acne prone skin and contribute to inflammation, to conventional antimicrobial therapies evoked the need to investigate new antimicrobial agents for the treatment of acne. Rosmarinic acid, being a phenolic compound obtained from *Rosmarinus officinalis,* shows an antimicrobial potential against many Gram-positive and Gram-negative bacteria [175].

Therefore, rosmarinic acid was incorporated into niosomes (Span^®^ 85 and cholesterol) and, further, into a carbomer gel, which was compared to the plain drug solution and marketed BPO gel [153]. As to the in vitro antimicrobial activity, the inhibition zone against *S. aureus* and *P. acne* revealed that niosomes and the niosomal gel demonstrated a better antibacterial activity profile in comparison to the plain gel. In vivo in Swiss albino mice, it has been shown that the niosomal gel provided a prolonged antimicrobial activity against *P. acnes* in contrast to the marketed gel and plain solution of rosmarinic acid (Table 3). As to the irritation potential studied in vivo in Swiss albino mice, the optimized niosomal gel formulation was significantly less of an irritant as compared to the plain rosmarinic acid solution and is considered to be safe for dermal application. Based on in vivo antiinflammatory studies, it has been shown that the rosmarinic-acid-loaded niosomal gel induced the higher inhibition of inflammation than the plain rosmarinic acid solution. The in vitro skin permeation and skin retention studies revealed that the rosmarinic-acid-niosomal gel led to a higher permeation, but also to a higher skin retention of rosmarinic acid compared to the plain gel (Table 3). Thus, the rosmarinic-acid-loaded niosomal gel, due to its high antimicrobial activity against *P. acnes* and ability to provide rosmarinic acid in the skin layers, presents a promising approach for treating acne vulgaris [153].

### 6.10. Tea-Tree-Oil-Loaded Liposomes

Tea tree oil, also known as melaleuca oil, is a monoterpene rich, lipophilic, essential oil derived by steam distillation from the Australian native plant *Melaleuca alternifolia* (Myrtaceae). The oil contains approximately 100 components, with the most abundant component terpinen-4-ol comprising approximately 40% of the oil. Tea tree oil has a broad-spectrum antimicrobial activity, and non-specific cell membrane damage is its major mechanism of antibacterial action. Several studies have shown that it reduces the number of lesions in patients with mild-to-moderate acne [176,177]. Tea tree oil has antibacterial, antifungal, and antiviral activities, due to the presence of terpinen-4-ol [178]. In addition, the oil possesses activity against biofilms of Gram-positive bacteria such as *Staphylococcus aureus*, suggesting that it may also affect *P. acnes* biofilms. It also shows anti-inflammatory activity, being a critical factor in the pathogenesis of acne [178]. Minor adverse effects observed in clinical studies, including pruritus, burning, stinging, scaling, itch, redness, and dryness, were consistent with those commonly associated with topically applied acne treatments. As to the skin penetration, tea tree oil penetrates poorly into and through human skin, since most of the oil is lost by evaporation, leading to low levels of tea tree oil components in the SC, but not in the deeper skin layers [179]. However, tea tree oil components have been demonstrated to be retrievable from follicular casts in bovine udder skin [180].

Its penetration into the skin could be enhanced, as well as its therapeutic efficacy in treating, e.g., acne, while reducing its adverse effects, by incorporating it into different nanocarriers, such as liposomes, sponges, nanofibers and films, etc. [181]. There have been efficient methods developed for tea tree oil encapsulation into liposomes, composed of PC and Chol (ratio 5.51), 1.21% (*v*/*v*) tea tree oil, and 0.79% (*v*/*v*) Tween 80 [182]. Liposomal formulations exhibited a constant rate of terpinen-4-ol release [181]. It has been shown that encapsulating tea tree oil (single and in combination with silver) and silver (as the ion Ag(+)) in a liposomal carrier system can improve their antimicrobial efficacy against *Pseudomonas aeruginosa*, *Staphylococcus aureus,* and *Candida albicans*, as well as decrease its concentration required for inducing therapeutic effectiveness [183]. A previous investigation involving tea tree oil encapsulated in liposomes, microemulsion, multiple emulsion, and other formulations revealed that the microbial activity of encapsulated tea tree oil is influenced by the pH value. Specifically, formulations containing 5% w/w tea tree oil exhibited the highest efficacy against *P. acnes*, *S. aureus*, and *S. epidermidis* at pH 5.5 [180]. These findings encourage the potential use of tea tree oil encapsulated in liposomes for the treatment of acne.

## 7. Photodynamic Therapy (PDT) Combined with Active Agents Encapsulated in Vesicles

Antibiotics have usually been used to treat acne, often in combination with keratolytic agents, which unfortunately leads to antibiotic resistance of *P. acnes.* Thus, there has been a search for new anti-acne drugs, as well as for new kinds of treatment. Photodynamic therapy (PDT) has been proposed as a treatment option for acne vulgaris. The main problem in topical PDT is the insufficient penetration of the photosensitizer (PS) into the skin, which limits its use to only superficial skin lesions. Liposomes could enhance the photosensitizers’ penetration into the skin, while decreasing its absorption into systemic circulation [184].

### 7.1. PDT and Methylene-Blue-Loaded Conventional Liposomes

Methylene blue (MB) was also encapsulated into liposomes, which were further formulated into a hydrogel (Table 3). The 0.1% methylene-blue-loaded liposomal hydrogel was investigated for its efficacy and skin tolerability in the PDT of patients with mild-to-moderate acne vulgaris in a randomized, controlled, and investigator-blinded study [158]. The release of the drug from the conventional MB-gel was considerably higher than that from the liposomal MB-gel. While the gel with free MB caused superficial destruction in the mice hair shaft, the liposomal MB gel resulted in the complete destruction of the pilosebaceous unit. In terms of treating acne, the liposomal gel demonstrated significant effectiveness with an 83.3% reduction in inflammatory acne lesions and a 63.6% reduction in non-inflammatory acne lesions after only two sessions. After prolonged treatment, i.e., after 12 weeks, 90% of patients exhibited a moderate-to-marked improvement in the treated areas. In terms of pain and local adverse effects, most patients experienced no pain, and no serious adverse side effects or edema were recorded. However, slight, transient hyperpigmentation was observed, but only in three patients. The authors illustrated that the liposomal MB-gel effectively delivered the drug selectively to sebaceous glands, the target site, making it successful in the PDT of mild-to-moderate acne vulgaris [158].

MB-loaded liposomes were also investigated in another study where their effect in combination with PDT was analyzed versus intense pulsed light (IPL) alone, in truncal acne vulgaris (of varying types) in 35 patients [159]. The protocol is explained in Table 3. The severity of acne was graded by the Burton scale, while patient satisfaction was evaluated by the Cardiff Acne Disability Index (CADI) before and after the treatment. The results revealed that, on the back side treated with the MB-liposomal gel, inflammatory acne lesion counts were significantly decreased compared to the treatment with only IPL (Table 3). Further, a significant overall improvement was seen on the back side, which was pretreated with the MB-liposomal gel. There was a correlation between the CADI score and overall improvement. The authors demonstrated that the pretreatment with the methylene-blue-liposomal gel followed by IPL was more effective than IPL alone, as well as safe with tolerable pain, in the treatment of acne vulgaris on the back [159].

### 7.2. PDT and Pheo-A-Loaded Elastic Vesicles

Park et al. have developed a new approach which relies on the use of PDT and the application of transfersomes, i.e., the *P. acnes* lipase-sensitive transfersomes (DSPE-PEG-Pheo A (DPP) transfersomes) (Table 3) [157]. These Pheo-A-loaded vesicles were developed in order to enhance the selectivity of drug activity, as well as to enhance the skin penetration of the drug, since Pheo A possesses unfavorable properties for skin penetration (very low water solubility and a high molecular weight of 592.68 Da). In vitro penetration studies in normal mice skin and *P. Acnes*-induced mice skin have shown that the DPP transfersomes significantly enhanced the skin penetration of Pheo A compared to that of Pheo A in DPP conventional liposomes and free Pheo A. Namely, in contrast to the Pheo A solution and Pheo-A-loaded liposomes, which delivered Pheo A to the SC or to all skin layers, respectively, the fluorescence of Pheo A from DPP transfersomes was only detected in the dermis of *P. acnes*-induced skin. This selective drug delivery to the dermis of *P. acnes*-induced skin was achieved by the increased deformability and hydrophilicity of the DPP transfersomes due to the added Tween^®^ 80 (edge activator enhancing vesicle flexibility and deformability) and PEG. Due to their deformability, transfersomes were able to squeeze between the intercellular spaces of the SC. Furthermore, the increased hydrophilicity of transfersomes, facilitated by PEG, reduces osmotic stress, allowing the transfersomes to convey the photosensitizer to the dermis of *P. acnes*-induced skin along the transepidermal water gradient. Due to their composition, transfersomes are sensitive to *P. acnes* lipase, and the photoactivity of Pheo A quenched by DPP transfersomes is gradually restored through the selective cleavage of the ester linkage by *P. acnes* lipases. In vitro studies demonstrated that *P. acnes*-specific photoactivity resulted in an over-99% reduction in *P. acnes* viability. Additionally, in vivo experiments using nude mice with induced *P. acnes* confirmed the therapeutic efficacy of transfersomes against acne, evidenced by a reduction in swelling volume and skin thickness. These findings highlight that DPP transfersomes achieve the targeted delivery of the incorporated photosensitizer Pheo A to the dermis, the primary location of *P. acnes*, enabling efficient targeted photodynamic therapy for acne and other bacterial skin infections [157].

### 7.3. PDT and 5-Aminolevulinic-Acid-Loaded Liposomes

Liposomal encapsulation of the photosensitizer 5-aminolevulinic acid (5-ALA), which is used for the PDT of acne, enabled a lower concentration than the standard concentration of 5-ALA to be used, which minimizes the risk of post-treatment photosensitivity. The use of 0.5% liposome-encapsulated 5-ALA spray with intense pulsed light reduced inflammatory lesions by 52% at 1 month and 65% at 6 months after the treatment of inflammatory facial acne, without significant side effects (Table 3) [161].

## 8. Conclusions

Nanotechnology has transformed dermato-pharmacotherapy, offering innovative solutions to address challenges in dermal drug delivery. Among various nanocarriers, liposomes have been extensively studied. They enhance drugs’ stability and their skin accumulation, thereby improving therapeutic effectiveness. Liposomes serve as drug reservoirs in the skin, enable controlled drug release, reduce required doses, and minimize side effects, such as skin irritation. This, in turn, enhances patient compliance. Consequently, liposomes and related vesicles are ideal carriers for anti-acne drugs, addressing challenges related to their insufficient skin penetration and poor skin tolerability, including dryness, peeling, and erythema. This increased skin tolerability contributes to enhanced patient compliance, a critical factor for overall therapeutic success.

Regarding the safety and biocompatibility of liposomes, it is noteworthy that phospholipid vesicles, primarily composed of phospholipids (PLs), are inherently biocompatible, biodegradable, and hold a Generally Recognized as Safe (GRAS) status. This assertion is substantiated by numerous studies discussed in this review, which consistently affirm the favorable safety profile of liposomes. Both in vitro and in vivo assessments have demonstrated no irritation potential associated with liposomes. Furthermore, the commonly used phospholipid in liposomes is non-hydrogenated soybean lecithin, characterized by the presence of essential fatty acids such as linoleic acid and linolenic acid. These essential fatty acids play a crucial role in maintaining the integrity of the skin barrier [185]. Linoleic acid has been utilized for addressing skin dryness and scaling by restoring the skin barrier function [185,186]. Moreover, linoleic acid has been implicated in the pathogenesis of acne due to its impact on sebocyte differentiation and its involvement in the disrupted keratinization of the follicular infundibulum [128]. The diminished levels of linoleic acid on the skin surface observed in acne patients may contribute to the initiation of hyperkeratinization, leading to subsequent comedogenesis [128,187]. The hypothesis suggests that the reduced concentration of linoleic acid in sebum induces a state of fatty acid deficiency in the cells of the follicular epithelium, resulting in abnormal differentiation and subsequent hyperkeratosis [188]. Additionally, when applied topically, linoleic acid exhibits comedolytic properties and has the potential to decrease the size of microcomedones [189]. Thus, linoleic acid applied to the skin, e.g., in the form of phospholipid liposomes, is expected to have beneficial effects on the skin of patients with acne.

Regarding other related vesicles, their safety profile depends on the properties of their constituents besides PL, such as different surfactants.

However, the practical application of liposome-based drug delivery systems in clinical settings has not advanced as rapidly as expected despite the promising outcomes observed in research. The current problem in the development of anti-acne drug-loaded carriers is that the majority of studies were conducted using in vitro and in vivo animal models, and normal skin. It is important to highlight that these follicles differ from those involved in acne, as they are closed owing to the presence of dry sebum and shed corneocytes. This closure may potentially impede the penetration of drugs into the follicles. Thus, more studies using skin with induced acne are needed, as well as more clinical studies, in order to obtain more accurate results.

Challenges in the development of liposomes and most liposome-related vesicles are limited physical stability due to vesicles’ aggregation, as well as chemical instability leading to the leaking of the cargo, which limit their shelf life and efficacy. Controlling liposomes’ size can also be a challenge, while it is often important for their efficacy. The encapsulation efficiency of drugs in the vesicles can also be low, which reduced the efficacy of the formulation. Further, there are scale-up challenges as producing liposomes on a large scale for clinical use can be complex and expensive, posing challenges for widespread adoption. Thus, the limitations in the pharmaceutical development of liposomes are focused mostly on quality assurance and cost. Quality assurance in the manufacturing of liposomes is focused on concerns related to both the manufacturing process and the stability of the formulation. These systems encounter challenges such as: (i) the scalability of the manufacturing process, (ii) the reliability and reproducibility of the final product, (iii) the absence of adequate equipment and/or in-house expertise, (iv) the potential for chemical instability or denaturation of the encapsulated compound during manufacturing, and (v) long-term stability issues [190]. Therefore, despite the plethora of encouraging results in research studies, as well as in preclinical studies, the clinical translation of liposomal drug delivery systems is slow.

These disadvantages of liposomes led to the development of other colloidal drug delivery systems, being used as efficient drug carriers for different delivery routes, such as solid lipid nanoparticles, nanostructured lipid carriers, polymeric nanoparticles, and other polymeric nanocarriers. These nanocarriers may overcome some of the drawbacks of liposomes, such as the high cost, potential vesicles’ instability, limited encapsulation efficiency, and others. However, when introducing polymeric- and surfactant-based nanocarriers, caution should be taken regarding the safety profile of the polymers and surfactants used in the formulation.

## 9. Future Directions

In the realm of acne treatments, the efficacy of liposomal encapsulation is principally attributed to the ability of liposomes to penetrate into the skin, selectively target hair follicles, and facilitate the controlled release of drugs. Additionally, liposomal encapsulation enhances the stability of drugs and minimizes skin irritation. These features also hold for other described nanocarriers. Given these insights, our perspectives are outlined as follows.

Future directions in the utilization of liposomes for acne treatment include the exploration of novel anti-acne drugs characterized by increased therapeutic efficiency and reduced skin irritation. Moreover, there is a focus on the development of innovative carriers, such as smart nanocarriers, specifically liposomes, that can respond to distinct stimuli, such as alterations in pH or temperature. These carriers enable the controlled and targeted release of their payload [191].

In addition, further investigations involving human volunteers are imperative, as existing studies predominantly rely on in vitro and in vivo assessments in animal models. Moreover, emphasizing the necessity to optimize the large-scale production of liposomes loaded with anti-acne drugs through a simplified methodology is crucial, as it plays a pivotal role in fulfilling clinical demand. Finally, the imperative consideration of regulatory aspects is essential prior to clinical implementation.

Through continued research endeavors, encompassing the introduction of new drugs, the development of smart liposomes, and the discerning findings from clinical trials, the potential of liposomes in dermatology, particularly in the context of acne treatment, remains highly promising.

## Figures and Tables

**Table 2:** Vesicles used as carriers for antibiotics.

Formulation	Drug	Therapeutic Indication	Experimental Condition	Outcome	Reference
**Antibiotics** **Clindamycine-loaded liposomes in clinical study**					
MLV * liposomes with 1% clindamycine, 1% clindamycine solution (Klimicin T^®^, Lek, Ljubljana, Slovenia)	Clindamycine	Acne vulgaris	In vivo, double-blind clinical study, 73 patients	The safety and efficiency of 1% clindamycine-loaded liposomes versus 1% clindamycine solution was investigated. Liposomes with 1% clindamycine were therapeutically superior over the conventional 1% clindamycine solution in the treatment of acne vulgaris, and no side effects were reported.	[119]
**Clindamycine-loaded liposomes**					
Five different liposome formulations were prepared (molar ratio of Phospholipon^®^ 85G (PL) and CHOL was varied). Into liposomes with optimal PL:CHOL ratio, charged lipids were added: 1,2-dioleoyl-3-trimethylammonium-propane chloride salt (DOTAP) and 1,2-dimyristoyl-sn-glycero-3-phosphate monosodium salt (DMPA) to obtain cationic or anionic liposomes. Molar ratio 0.5 of CHOL to PL was optimal.	Clindamycine-phosphate	Acne vulgaris	In vitro, mice skin	The steady state flux of the drug was 4.4-fold higher when it was encapsulated in cationic liposomes compared to the control (drug dissolved in mixed alcohol solution) and approx. 1.4-fold higher than when it was encapsulated in anionic and neutral liposomes. Cationic liposomes were the most efficient delivery system.	[120]
Transfersomes (PC:Span^®^ 80) were incorporated into a carbomer gel and compared to control carbomer gel	Clindamycine-phosphate	Acne vulgaris	In vitro release	Clindamycine phosphate showed a significantly higher in vitro release from the transfersomal gel compared to the control carbomer gel.	[121]
			Ex vivo, rat skin	Transfersomal gel showed a significantly higher drug permeation though the skin and flux compared to transfersome dispersion and control carbomer gel, which provided higher penetration and deposition of clindamycine in the skin.	
**Tetracycline-hydrochloride-loaded liposomes**					
**Liposomes, transfersomes, aqueous solution**	Tetracycline hydrochloride	Acne vulgaris	*Staphylococcus epidermis*	MIC values did not differ between vesicles and solution of the drug.	[122]
			In vitro drug release	Liposomes released a 2.6-fold-higher tetracycline hydrochloride amount than transfersomes, but a significantly lower amount than the drug solution, indicating prevention of drug leakage, until it is delivered to the target tissue and micro-organism.	
			Ex vivo drug permeation, rat skin	Transfersomes and liposomes increased skin deposition of the drug by 3.2- and 1.9-fold, respectively, compared to the tetracycline hydrochloride aqueous solution. In contrast, transfersomes delivered lower amounts of drug to the receptor phase than liposomes, being optimal for treating skin diseases.	
Liposomes containing two drugs were incorporated into a carbomer gel.	Tetracycline hydrochloride and tretinoin	Acne vulgaris	In vitro release	Liposomal gel provided a sustained drug release for 24 hours with released amounts of the following: Tetracycline hydrochloride: 56.44% Tretinoin: 58.44%	[123]
			Cytotoxicity test, in mice fibroblast cells	The cytotoxicity test confirmed that the liposomal gels were non-toxic.	
			Antimicrobial test in *Staphylococcus aureus* and *Streptococcus epidermidis* strains	Antibacterial efficacy of liposomal gels has been confirmed.	
**Roxithromycin-loaded vesicles**					
Niosomes, proniosomes, ufosomes, and pro-ufosomes	Roxithromycin in different solid-state forms (i.e., crystalline and amorphous)	Acne vulgaris	In vitro	All vesicles delivered the drug to the dermis, while no drug was found in the receptor compartment and stratum corneum–epidermis part of skin. Niosomes were the best formulation, and amorphous forms achieved higher topical delivery.	[124]
**Dapsone-loaded liposomes**					
Invasomes	Dapsone	Acne vulgaris	In vivo, Wistar rats	Invasomes delivered a 2.5-fold-higher drug amount to the skin compared to the drug solution.	[125]

* MLV—multilamellar vesicles.
